# Lip reading systems for Urdu alphabets in diverse environments

**DOI:** 10.1038/s41598-026-53299-5

**Published:** 2026-06-14

**Authors:** Amanullah Baloch, Mushtaq Ali, Lal Hussain, Ali Daud, Hussain Dawood

**Affiliations:** 1 Department of Computer Science & Information Technology, Hazara University Mansehra, Mansehra, 21120 Pakistan; 2 Department of Computer Science, Neelum Campus, The University of Azad Jammu and Kashmir, Athmuqam, 13230 Pakistan; 3 Faculty of Resilience, Rabdan Academy, Abu Dhabi, United Arab Emirates; 4 School of Computing, Horizon University College, 5700, Al Ajman, UAE; 5 Jadara University Research Center, Jadara University, 21110, Irbid, Jordan

**Keywords:** Lip reading system, Urdu alphabets dataset, DNN models, Data augmentation, Confusion matrix, Engineering, Mathematics and computing

## Abstract

Lip reading technology has potential use across various fields, significantly enhancing communication for the deaf, aiding in noisy settings, and supporting information security through silent password entry. Although, notable progress has been made in constructing datasets of different types like digits, alphabets, words, phrases, and sentences levels for lip reading in various languages. However, developing a robust Urdu lip reading model remains a challenge due to the lack of a suitable dataset. Moreover, difficulties in adapting previous models, such as the LipNet model to Urdu. To address these barriers, we present the ULRA (Urdu lip reading alphabets) dataset, leverage advanced data augmentation techniques, and evaluate three cutting-edge DNN models: a LipNet-based 2D-CNN model, a Hybrid 2D_3D-CNN model, and a baseline 3D-CNN model. Each model undergoes rigorous testing in diverse environments, with both familiar and unfamiliar data. The results reveal that the LipNet-based 2D-CNN model achieves an impressive 81.97% accuracy on unknown data across diverse environments, while the Hybrid model excels in generalization, reaching 69.45% accuracy on unfamiliar data, thanks to its superior spatiotemporal feature extraction capabilities. Additionally, precision, recall, and F1-Score values of LipNet-Based 2D CNN are 0.83, 0.82, and 0.82 respectively. All three values of this model are also higher than the other two models. These findings underscore the strengths of various DNN architectures and the critical advancements made possible by the ULRA dataset, paving the way for future breakthroughs in Urdu lip reading research.

## Introduction

**Fig. 1:**
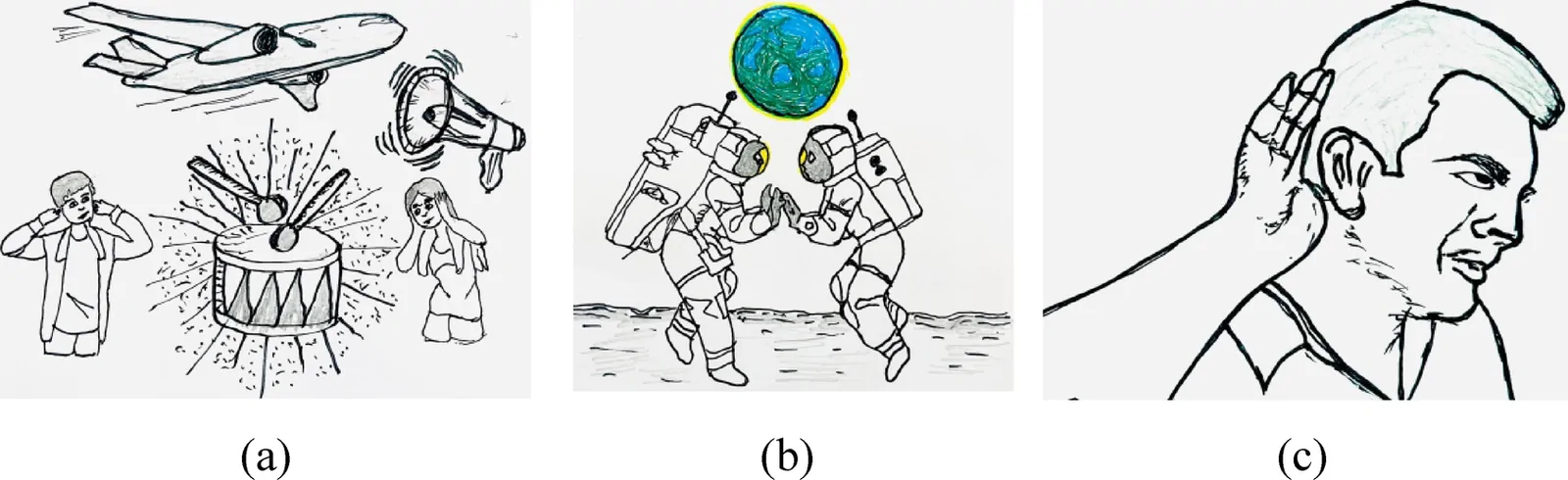
Applications of lip-reading system. (**a**) people missing parts of speech due to noise, (**b**) astronauts in voiceless environment, (**c**) deaf community.

 Lip-reading refers to the ability to interpret a speaker’s words by observing the movement of their lips. In computing, lip reading can be particularly useful in noisy environments to reconstruct disrupted speech signals^1,2^, as depicted in Fig. 1(a). Additionally, in space, where sound cannot travel due to the absence of a medium, lip reading can aid astronauts in communicating through lip movements alone, as shown in Fig. 1(b). This technology also has significant applications for the deaf, allowing text to be extracted from a speaker’s lip movements, as illustrated in Fig. 1(c)^1,3–19^. For instance, video lectures and political speeches can be converted into text for the deaf, enabling access to spoken content. Furthermore, two deaf individuals could communicate with each other, much like astronauts, without relying on hand signals or spoken language. Lip reading can also enhance interactions between the hearing and deaf communities, allowing smoother communication without the need for sign language, which has traditionally been a barrier for those unfamiliar with it^19,20^.

In addition to the previously mentioned applications, lip reading can be used to securely input silent passwords in crowded environments by relying on lip movements^6,21^. Moreover, biometric identification systems can be fooled by presenting a photograph of a person to the machine^1^. However, lip reading provides a more robust solution, as it requires multiple image frames in the form of a video clip to identify a person through a spoken code word, rather than a single picture^13^. Additionally, lip reading can assist in recovering videos where sensory agencies have muted the audio from certain objectionable parts of a speaker’s speech.

To address the challenges mentioned above, numerous efforts have been made across different languages. Researchers have developed lip reading datasets in their respective languages to advance this field, including English^1–4,9,11^, Turkish^6,7^, Thai Chinese^10^, Chinese^12^, Arabic^22–24^, and Hindi Devanagari^25^. These datasets are available for researchers working on lip reading and encompass various categories such as digits^10,12^, alphabets^24,26,27^, words^5,6,12^, phrases^5^, and sentences^3,4,6,9,11^, in multiple international languages.

All the aforementioned efforts have been made at various levels in different languages. For instance, some languages have focused on digits, while others have concentrated on alphabets. Attempts at the word and phrase level have also been made in certain languages, with more recent efforts advancing to sentence-level lip reading. However, when it comes to the Urdu language, there has been only one attempt, which was unsuccessful, made by^5^. This paper did not introduce a new model but instead adopted the LipNet model from^3^ for Urdu sentence-level lip reading. The author claimed to provide a link to an Urdu dataset in the article^5^, but the provided URL was inaccessible. Moreover, the performance of the LipNet model on this referenced Urdu dataset was unsatisfactory.

In addition^5^, acknowledged that they were unable to properly train the model for Urdu words and sentences, resulting in outputs of fixed-length six-word sentences that were neither meaningful nor aligned with the ground truth, such as “How are you” and the Roman Urdu word “Shuru”. The accuracy of the Roman Urdu output was also poor. The reason for these unsatisfactory and illogical results lies in the fact that the adopted LipNet model was designed for the GRID corpus, where each sentence consists of six words. However, the dataset created by^5^ did not align with the LipNet model’s design. Despite the potential benefits of lip reading technology for hearing-impaired individuals and silent communication, no successful Urdu lip reading system has been developed due to the lack of a suitable dataset and the failure of prior models like LipNet for Urdu^38^. presents the first successful Urdu lip reading system by introducing the ULDR dataset of 2,100 digit video clips and comparing three deep neural network models, achieving up to 92.15% accuracy.

Although, there are 230 million people in the world who speak Urdu^28^, no successful efforts have been made to develop an Urdu lip-reading system. The Urdu language is a sequence of characters and uses the sequential model of CNN^28–33^ Likewise the video frames used in lip-reading are the sequence of frames, and also use the sequential model. There is an analogy that both tasks use pattern recognition and sequence modeling. The lack of a dedicated Urdu lip reading dataset has been a significant obstacle for researchers interested in this field, as the availability of such a dataset is crucial for progress in Urdu lip reading research.

The objectives of this research work are described as follows.

(i) To develop Urdu Lip Reading Alphabets (ULRA) dataset, consisting of 8,400 labeled video clips. (ii) Prepare the dataset through various preprocessing steps, including video frame separation, mouth region extraction, resizing of the mouth section, padding frames that fall short of the required limit, and image enhancement. (iii) To propose and implement three DNN model architectures for a comparative analysis of Urdu letter recognition. (iv) To identify best Urdu Lip Reading model among three DNN models based on letter level misclassification.

The research questions related to the aforementioned objectives are given bellows.

(i) How Urdu Lip Reading Alphabets (ULRA) dataset is to be developed? (ii) What preprocessing steps are applied for the preprocessing of ULRA dataset? (iii) How to select the best DNN model architectures among the three DNN models?

The remainder of the paper is structured as follows: Sect.  2 provides a detailed discussion of related work. Section  3 introduces the proposed work, including the three architectural models. In Sect.  4, the results are presented and discussed. Finally, Sect.  5 concludes the research work.

## Related work

In this section, the relevant literature is decomposed into different categories of lip-reading datasets built by researchers in different languages.

### Digits lip-reading datasets

In^10^, a method to enhance lip-reading recognition by concatenating three sequential keyframes is introduced. This technique was implemented using two datasets: AVDigits and THDigits. AVDigits is a small dataset consisting of ten digits, recorded by six speakers, with each digit spoken nine times per speaker. The dataset includes 10 digits in total. In contrast, THDigits was recorded by 100 male and female speakers. The video frames from both datasets underwent preprocessing, where the lip region was cropped and resized to 32 × 32 pixels. The training and testing data were split into 83% and 29%, respectively. Model performance was evaluated using accuracy and cross-entropy. Extracted lip features were fed into the model’s classifier, which activated one of the ten possible outputs. Six methods MDBN, MDAE, RTMRBM, AM-LSTM, AM-GRU, and the proposed C3-SKI were tested on the AVDigits dataset. The proposed model outperformed the other five. Additionally, when tested on the THDigits dataset, it achieved even better accuracy than on AVDigits.

In^12^, a hybrid lip-reading model incorporating the convolutional block attention module (CBAM) and ResNet50 was proposed. A self-constructed Chinese dataset, consisting of ten digits and ten commonly used words, was developed for training and evaluation. Deep neural networks often encounter gradient vanishing issues during backpropagation. ResNet50, an advanced neural network, addresses this problem and maintains model stability. The architecture consists of five components. First, video clips are preprocessed, with keyframes selected for feature extraction. Ten frames from each video clip are input into the model. The second component, ResNet50 combined with CBAM, extracts lip features while preserving weights. CBAM enhances feature extraction through two steps: (1) The channel module emphasizes important content, and (2) The spatial module refines information by processing each pixel. A sigmoid function combines these two steps into channel attention weights. The output is then passed to a GRU module, which handles long-range dependencies and maintains contextual understanding. An attention mechanism further boosts accuracy by assigning importance to key lip sequences. Finally, the output layer classifies the results. The dataset was built with 50 speakers, each pronouncing 10 digits and 10 words ten times, resulting in 10,000 video clips. These were divided into 8,000 for training and 2,000 for testing. Six state-of-the-art models were compared, including LSTM + attention and GRU + attention with VGG16, InceptionV3, ResNet50, ResNet101, ResNet152, and the proposed ResNet50 + CBAM. The proposed model achieved the best performance across all evaluation criteria.

In^38^, first successful Urdu lip reading system is presented, it addresses the lack of an appropriate dataset and the failure of prior models like LipNet for Urdu. The authors introduce the ULDR dataset, containing 2,100 video clips of 10 Urdu digits spoken by 15 speakers in both controlled (indoor) and uncontrolled (outdoor) environments, augmented sixfold using techniques such as translation, flipping, rotation, color jittering, and blurring. They compare three deep neural network models: a Hybrid 2D-3D CNN-LSTM, a LipNet-based 2D CNN-LSTM, and a 3D CNN-GRU. The LipNet-based 2D CNN-LSTM model achieves the highest overall accuracy (92.15%) across all conditions, with precision, recall, and F1-score of 0.91 each. The hybrid model demonstrates strong generalization on unseen data (90% accuracy), while the 3D CNN-GRU model shows overfitting. The study also finds that horizontal flip and + 10° rotation augmentations degrade performance and should be avoided.

### Alphabets lip-reading datasets

In^24^, Arabic alphabets and word lip-reading systems based on their own AQAND, Al-Qaida Al-Noorania Dataset is presented. The dataset consists of 10,490 videos of three categories, 29 single letters, 14 disjoint letters, and 10 Quranic words. Videos were recorded from three different angles 0^o^, 30^o^, and 90^o^ in a control environment. The dataset was divided into 7630, 1430, and 1430 videos for training, validation, and testing respectively. The two augmentation techniques, horizontal flip, and affine transformation are applied on the training and validation part of the dataset. Each augmentation technique contributed 7630, and 1430 videos by extending the training and validation part of data up to 22,890, and 4290 videos. A lip-reading deep learning model based on ResNet-18 was trained and tested using AQAND datasets. The lip reading system gave 77.5%, 80.5%, and 83.3% accuracies on single letters, disjoint letters, and Quranic words respectively.

In^26^, an alphabets-based lip motion recognition using a neural network is proposed. The dataset comprised 10 speakers, each speaker spoke 26 English alphabets. Each alphabet is recorded as a video clip of 20 frames. Lips of each frame of the video clip are segmented based on differentiating skin color pixels of lips and surrounding skin. Nearly nine geometrical mouth shape features are extracted. These features are stored in the database to train the neural network model so that the model can classify and predict the tested video for the required letter. In this research, 65% accuracy on English alphabets is achieved.

In^27^, a Filipino speaker English letter pronunciation-based lip reading analysis is proposed. The training part of the dataset consists of the videos of 30 Filipino speakers, each pronouncing 26 English alphabets two times i.e., the total numbers of training videos were 1560. The testing dataset includes 10 Filipino speakers, each pronounced 26 English alphabets only once i.e., the total number of testing videos was 260. Three models based on 8, 12, and 16 geometrical features of lips sections were trained using traditional decision tree methods. The performance of these models was measured on test data. It was observed that a model based on 16 geometrical features shows a high accuracy of 45.16%.

### Words lip-reading datasets

In^6^, a Turkish lip-reading dataset was developed. Traditional machine learning methods had limited success in advancing lip-reading research, but deep neural networks significantly improved the field. One major challenge is the lack of datasets in various languages. The authors of this study addressed this gap by creating two Turkish datasets: (i) a word dataset with 111 frequently used words and (ii) a sentence dataset with 113 common phrases. Twenty-four speakers participated in recordings, captured from 1.5 m using an iPhone 11 with a 12-megapixel camera at 30 and 60 fps. To account for slight head movements, video clips recorded at angles from + 10 to -10 degrees were corrected using image processing techniques. Eleven state-of-the-art neural networks were trained and tested on both datasets, with an 80 − 20 train-test split. ResNet-18 and GoogleNet, combined with Bi-LSTM, achieved the best results. GoogleNet attained 69.6% and 72.33% accuracy on the word and sentence datasets, while ResNet-18 achieved 84.09% and 88.55%, respectively.

In^7^, a dataset for the Turkish language is presented. It includes five Turkish words and five phrases. Their English translations are as follows: start, finish, hello, good morning, hi, and welcome, sorry, see you, enjoy your meal, and thank you. Unlike datasets recorded in controlled environments, the video clips in this dataset were sourced from TV series, movies, vlogs, and songs. Since the speakers were not specifically recruited for recording, special attention was given to ensuring a balanced number of videos for each word and phrase. The dataset contains a total of 2,335 instances, with an average of 234 instances per word or phrase. It is organized into ten folders, each named after the corresponding word or phrase, containing subfolders with video clip instances categorized accordingly. Each subfolder further includes image files representing video frames corresponding to the relevant words or phrases.

### Phrase lip-reading datasets

In^5^, the LipNet model from^3^ was adapted for Urdu sentence-level lip reading. A dedicated Urdu dataset was created for training, featuring recordings from ten male and female speakers. However, the referenced dataset URL in^5^, is no longer accessible via Google. The LipNet model failed to produce satisfactory results in Urdu, showing limitations in handling Urdu words and sentences. The model’s fixed six-word output format did not align with the flexible structure of Urdu sentences. Additionally, its Roman Urdu predictions were inaccurate. This issue arose because the original LipNet was designed for the GRID corpus, where each sentence consists of six words, whereas the dataset in^5^ did not follow this structure.

### Sentence lip-reading datasets

In^3^, an end-to-end lip-reading model for sentence-level recognition was introduced. Both male and female speakers participated, each recording 1,000 sentences, resulting in a dataset of 34,000 video clips. The model was trained on 3-second video clips containing 75 frames. It was evaluated on two scenarios: overlapped and non-overlapped speakers. The word error rate (WER) and character error rate (CER) were lower for overlapped speakers. The model was trained on the GRID corpus, where all video clips featured speakers in a frontal pose, captured in a controlled environment using high-quality cameras.

In^4^, a hybrid deep CNN-based lip-reading model was developed, also trained on the GRID corpus. The model consists of three stages: preprocessing, encoding, and decoding. The preprocessing stage follows the same steps as in^3^ to isolate the lips. The encoder processes 75-frame video clips, extracting features for the decoder. The attention layer applies a dot product between the encoded lip movement features and bidirectional RNN attention weights. The output is then processed by the CTC model, which arranges characters into words and words into sentences. This model incorporates several techniques from LipNet in^3^.

In^9^, a novel transformer-based lip-reading architecture was introduced, replacing CNNs and RNNs with a self-attention mechanism-based transformer network, originally proposed by Google in 2017. The model has three components. First, each frame from a 3-second video clip in the GRID corpus is cropped to a 60 × 120 × 3 lips region. Second, VGG16 extracts features from these cropped frames. Third, a transformer processes the extracted features, improving efficiency by handling them in parallel rather than sequentially like RNNs or CNNs. The transformer includes encoder and decoder layers, focusing on sentence context rather than merely selecting relevant text as LSTM and GRU do. The encoder tokenizes words, and the decoder assigns positions within sentences. The model achieved 45.81% accuracy in word-level lip reading.

In^11^, a lip-reading model trained on “Lip Reading Sentences in the Wild” (LRS) was introduced, using the “Watch, Listen, Attend, and Spell” (WLAS) network. The dataset comprises 100,000 sentences from BBC TV recordings of 500 speakers, rather than recordings made specifically for LRS. The model includes two encoders and a decoder. First, video frames are preprocessed, with lips cropped to 120 × 120 pixels. The “watch” encoder processes these frames, while the “listen” encoder handles corresponding audio input. The LSTM-based network then integrates the information.

In^1^, the preprocessing stage of a lip reading system is described. Video clips of words are split into individual frames, from which the mouth region is extracted using a Haar Cascade classifier. The edges of the mouth region are detected, and lip contours are identified. The extracted lip contours are then fed into a CNN for visual feature extraction, followed by a bidirectional GRU to model temporal dependencies across frames. Finally, a softmax layer classifies the words. The model was trained on the LRW dataset, which consists of video clips from BBC TV broadcasts, achieving a word accuracy of 90%.

In^2^, a multi-dilation temporal convolutional network model is introduced. This model employs a combination of convolutional layers followed by a self-attention block. This integration enhances the model’s classification ability to accurately predict individual words. The model was trained on the LRW dataset, which consists of English video clips, and achieved an accuracy of 85.7% in word recognition.

In^22^, a real-time Arabic viseme recognition system was developed. The system segments the lips from video frames by distinguishing their color from the rest of the face. This segmentation approach enables real-time identification of lips, improving the speed of the viseme recognition process. The extracted visual features of the segmented visemes are processed using a CNN classifier for classification. The model was trained on the SAVE dataset and achieved an accuracy of 95.3%.

In^23^, an Arabic lip-reading system called “Read My Lips” is introduced. This work contributed to the field by developing an Arabic word dataset for lip-reading research. Three different deep neural network (DNN) architectures were designed and evaluated to determine the dataset’s accuracy level. The system achieved an overall accuracy of 82.84%.

In^25^, an LSTM-based approach for lip reading in the Hindi (Devanagari) language is proposed. The dataset used for this study consists of a paragraph containing ten sentences (58 words) in Devanagari, spoken by 50 individuals (students and faculty members) from KLETech University. The system preprocesses video clips of each speaker by converting them into frames using OpenCV (cv2) functions. Facial landmarks, specifically the lips, are detected using the dlib library. The lip region in each frame is cropped, and the extracted features are passed through an LSTM layer to identify patterns through sequential analysis. These patterns are then fed into a dense layer, where each node represents a unique word in the dataset. Finally, the softmax layer classifies the predicted words or sentences. The model’s accuracy was recorded as 35–70% for word-level recognition and 20% for sentence-level recognition.

In^8^, a word-level Tibetan lip-reading dataset is introduced. The dataset consists of the 50 most frequently used Tibetan words. Twenty college students, both male and female, were selected as speakers for video recordings. Each speaker uttered each word three times, maintaining a 30 cm distance from the camera. The recorded videos had a resolution of 720 × 1280 pixels. The original videos were then compressed using the H.263 and H.265 codecs, creating additional copies encoded at 15 FPS and 25 FPS. To assess dataset quality, ten Tibetan-speaking college students evaluated the videos. Evaluators were given two hours to familiarize themselves with the annotated lip-reading clips. Out of 3,000 original video clips, each evaluator reviewed 500 randomly selected samples. Their comprehension was rated on a five-point scale: “Don’t understand,” “Relatively vague,” “Slightly understand,” “Basically understand,” and “Can understand,” with scores ranging from 0 to 5. The Mean Opinion Score (MOS) method was used to compute individual evaluation scores, resulting in an average score of 3.8 points per evaluator.

In^13^, a visual speech recognition system using CNNs in the VGG-M model is proposed. This study developed a physical security system based on lip reading using CNN technology. The model was trained on a self-created dataset consisting of digits (0–9) spoken by 15 speakers. It was observed that traditional biometric security systems using facial recognition are vulnerable to deception, as unauthorized individuals can bypass them using images of authorized persons. In contrast, the proposed model recognizes a person by analyzing sequences of image frames, ensuring security. The system achieved an accuracy of 87% on seen data and 30% on unseen data. However, since the system was designed to recognize only authorized employees or workers of an organization, the model effectively fulfilled its purpose.

In^14^,, a method for improving machine-based lip reading by identifying and classifying phonemes is presented. A phoneme-to-viseme mapping is created to classify visemes effectively. The dataset was recorded in a controlled environment and consists of 12 speakers, each uttering 200 samples across 1,000 English words. The study identified 45 viseme classes, with the smallest class containing eight phonemes. The findings highlight that viseme-based classifiers are more efficient than phoneme-based classifiers because visemes represent a smaller, more manageable set of units.

In^15^, a comprehensive survey on the advancements and challenges in deep lip reading is conducted. The survey is divided into three key sections: (1) the structure and development of datasets, (2) visual feature extractors, and (3) temporal feature extractors and sequential classifiers. The text datasets are categorized into five groups: characters, digits, words, phrases, and sentences. The study presents a hierarchical summary of the evolution of visual feature extractors from 2015 to 2020 and a tree-structured overview of temporal feature extractors from 2015 to 2021. The need for large, well-labeled datasets is emphasized to improve the accuracy of lip-reading models. Additionally, it is observed that visual features alone may not be sufficient to fully interpret speech. The integration of audio and textual information can enhance the accuracy of lip-reading systems when predicting test data.

In^16^, a novel method for enhancing lip reading through the distillation of speech recognizers is introduced. The system’s architecture consists of two key modules: the student module, responsible for extracting features from lip reading images, and the teacher module, which synchronizes these image features with speech features. Knowledge distillation occurs between these modules at three distinct levels: (1) frame-level, (2) sequence-level, and (3) context-level knowledge distillation. This multi-level temporal feature extraction, coupled with cross-verification between the two modules, significantly enhances system performance. The model was trained and evaluated on two prominent datasets, CMLR and LRS2, achieving character error rates of 31.27% and 45.53% on CMLR and LRS3, respectively.

In^17^, a comprehensive survey of lip reading techniques and datasets is presented. The survey explores deep learning approaches in lip reading and examines datasets across multiple languages used in research between 2017 and 2020. The study highlights the impact of RNN, CNN, and LSTM advancements in the field. It is observed that most available datasets focus on the English language, with a few covering Chinese, Malay, and Turkish. The accuracy of lip reading can be further improved by constructing datasets in uncontrolled environments with multiple viewing angles, as side-angle lip movements enhance recognition. Additionally, the study underscores the need for language-specific datasets to foster progress in lip reading research within diverse linguistic communities.

In^18^, a multi-grained spatiotemporal feature perception network (MSTP) for event-based lip reading is proposed. This model utilizes video clips captured at two different speeds using event-based cameras. Low-speed recordings offer superior spatial features but weak temporal details, whereas high-speed recordings provide strong temporal but weaker spatial features. The proposed approach extracts spatial features from low-speed videos and temporal features from high-speed recordings, merging them using the message flow module (MFM) to generate multi-grained spatiotemporal representations. The output of MFM is then processed by the sequence module to predict text based on lip movements. Since no suitable dataset was available, dynamic vision sensor (DVS) cameras were used to record data, and MSTP was compared with other event-based models, demonstrating superior performance.

In^20^, a dataset creation methodology is discussed, applied to the GRID dataset comprising video clips from 33 different speakers. The technique involves converting video clips into frames using the scikit-video library. Subsequently, face detection is performed using the dlib library, and the region of interest (ROI) containing the lips is extracted. To enhance the dataset, various image augmentation techniques—such as mirroring, noise addition, brightness adjustment, darkening, and rotation at different angles—are applied to the extracted lip images.

In^19^, a lip recognition algorithm integrating MobileNet with a GRU network and an attention mechanism is introduced. The system was trained on a small dataset containing digits from zero to nine. Due to its lightweight architecture, the model demonstrated reasonable performance, though its applicability was constrained by the limited scope of the dataset.

In^34^, a lip-reading using CNN with and without the pre-trained model in presented. AvLetters dataset with 10 speakers, each recorded every letter three times, i.e., two times for training data and one time for testing data. It means 65% of the dataset was reserved for training while the other 35% were used for testing purposes. The lip reading model was based on CNN architecture. Moreover the two pre-trained models i.e. AlexNet and GoogleNet were also trained and tested on the same dataset for comparison. To improve the performance rate, the size of the dataset is increased using different data augmentation techniques i.e., adding noise (pepper and salt), sharpening, and softening effects by applying various filters. The dataset was very small, the model was trained on only alphabets, and the model will down its performance on words and sentence level lip reading data.

In^35^, a deep learning-based content recognition model for lip reading is developed. The dataset comprises only 20 visual alphabetic letters. It was observed that six alphabet pairs—(A, E), (P, B), (F, V), (Q, W), (K, C), and (S, X)—share similar visemes and geometric shapes, rendering their separate storage unnecessary. The deep neural network (DNN)-based model incorporates multiple convolutional and max-pooling layers to capture fine-grained lip image features. The system enhances recognition accuracy for individual letters, though it was not extended to word- or sentence-level lip reading.

In^36^, a CNN-based lip reading model utilizing feature fusion is proposed. The model was trained and tested on the GRID and Oulu VS2 datasets. The CNN operates in two ways: one part employs the Histogram of Oriented Gradients (HOG) method to extract spatial features, while the other utilizes a bidirectional rank pooling technique for temporal feature extraction. The two feature sets are then fused and passed to a classifier for recognition.

In^37^, a deep learning-driven speech enhancement framework incorporating lip reading is presented. The system, trained on the GRID dataset, enhances speech recognition in noisy environments by combining noisy audio features with visual lip features, leveraging a multimodal deep learning approach.

In^28^, a comprehensive overview of the advancements, challenges, and future directions of Deep Learning (DL) and Natural Language Processing (NLP) is provided. It explores various DL architectures such as convolutional neural networks (CNNs), deep belief networks (DBNs), recurrent neural networks (RNNs), and long short-term memory (LSTM). The study discusses key NLP tasks, including speech recognition, machine translation, text classification, and named entity recognition, highlighting the role of word embeddings and large language models (LLMs). Additionally, the article delves into the limitations of existing DL approaches, such as data quality issues, lack of interpretability, and the difficulty of handling pragmatic aspects of language. Emerging trends such as few-shot learning, multimodal learning, and the need for more research in under-resourced languages like Urdu are also emphasized​.

In^29^, the development of an Urdu part-of-speech (POS) tagging system using Conditional Random Fields (CRF) is discussed. POS tagging is a fundamental task in Natural Language Processing (NLP) that assigns syntactic categories to words in a text. The study highlights the challenges of Urdu POS tagging, such as morphosyntactic ambiguity, and compares CRF with Support Vector Machines (SVM), the previously dominant approach. The CRF model outperforms the SVM-based model, achieving an 8.3–8.5% improvement in the F-measure. The study also introduces a balanced feature set, incorporating both language-independent and language-dependent attributes, and evaluates the model on benchmark Urdu datasets​.

In^30^, the application of machine learning and deep learning models for part-of-speech (POS) tagging in the Urdu language is explored. It compares various approaches, including Conditional Random Fields (CRF), Support Vector Machines (SVM), Hidden Markov Models (HMM), and deep recurrent neural networks (DRNNs) such as Long Short-Term Memory (LSTM) with and without CRF output. The study evaluates these models on benchmark Urdu datasets and highlights the challenges associated with POS tagging in Urdu, such as its rich morphology, lack of capitalization, and free word order. The results indicate that CRF outperforms other methods on the CLE dataset, while LSTM-based models show better performance on the BJ dataset​.

In^31^, the development of a semantic information retrieval (IR) benchmark for the Urdu language, following the guidelines of the Text retrieval Conference (TREC) is presented. The study addresses the scarcity of resources for Urdu IR by constructing the largest-ever Urdu news document corpus, comprising 2,887,169 documents scraped from 11 different newspapers. The authors generated 105 queries, including 35 original queries and two variations of each, to evaluate the effectiveness of IR techniques. A pooling-based approach was employed to identify candidate documents for manual relevance assessment, which was performed on a four-point scale (irrelevant, marginally relevant, fairly relevant, and highly relevant) by two experts. The benchmark aims to facilitate the evaluation of existing and future Urdu IR techniques, promoting semantic readiness and scalability in Urdu language processing.

In^32^, a comprehensive survey of Urdu Language Processing (ULP), highlighting the challenges and advancements in this field is provided. It discusses the scarcity of linguistic resources for Urdu, a widely spoken South Asian language, and compares it with resource-rich Western languages. The paper covers various aspects of ULP, including available datasets, characteristics of Urdu, pre-processing tasks like stop word removal and stemming, and core NLP tasks such as tokenization, part-of-speech tagging, named entity recognition, and parsing. It also explores the development of Urdu WordNet and its applications in information retrieval, classification, and plagiarism detection. The article concludes by identifying open issues and future directions for ULP research, emphasizing the need for large annotated datasets and improved statistical techniques.

In^33^, the development of a Named Entity Recognition (NER) dataset for Urdu, a resource-scarce language, to facilitate research in automated NER tasks using machine learning (ML) techniques is presented. The dataset, referred to as UNER, contains approximately 48,000 words with 4,621 named entities across seven categories: PERSON, LOCATION, ORGANIZATION, DESIGNATION, NUMBER, DATE, and TIME. The dataset is sourced from BBC Urdu and covers national, international, and sports news domains. The authors emphasize the importance of such datasets for training and testing ML models like Hidden Markov Models (HMM), Conditional Random Fields (CRF), and Recurrent Neural Networks (RNN). The dataset is freely available for research purposes and aims to promote Urdu NER research, particularly in the context of ML-based approaches.

Despite significant progress in visual speech recognition through deep neural networks, lip-reading research across international languages remains constrained by several fundamental gaps. A primary limitation repeatedly highlighted in the literature is the unavailability of large, standardized, and language-specific lip-reading datasets, which severely restricts the development and fair evaluation of robust models. As demonstrated in^6^, the lack of Turkish datasets necessitated the creation of new word- and sentence-level corpora to enable effective experimentation, underscoring how dataset scarcity remains a major obstacle for researchers working in under-resourced languages. Furthermore, linguistic diversity introduces additional challenges, as each language differs in character set size, phoneme–viseme mappings, and articulation patterns, resulting in a varying number of output classes across languages. Consequently, lip-reading models cannot be directly transferred between languages without architectural modifications. These variations affect both the input stage, where frame resolution and mouth ROI dimensions must be adapted to language-specific articulation characteristics, and the output stage, where the classification layer must be redesigned to accommodate the target language’s character inventory. Such dependencies lead to a lack of generalizable, language-agnostic lip-reading frameworks, forcing most existing studies to remain language-specific. Collectively, the scarcity of datasets, coupled with language-dependent architectural requirements at both input and output levels, represents a significant research gap in the current lip-reading literature.

## Proposed Lipnet-based 2D-CNN model for Urdu alphabets lip reading

The proposed work consists of the following sub-sections. (i) Data Preprocessing, (ii) DNN Models for Urdu Alphabets Lip Reading (a) LipNet-Based 2D-CNN Model (b) Hybrid 2D_3D-CNN Model (c) 3D-CNN Model, and iii) Construction of ULRA (Urdu Lip Reading Alphabets) dataset.

### Data pre-processing

The video clip inputs, provided to DNN Urdu lip reading models are first preprocessed by performing the following steps.

### Frames separation

**Fig. 2:**
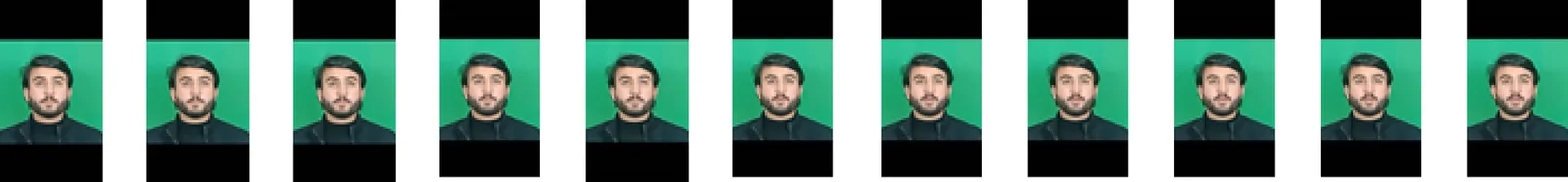
Original frames of the video clip, The speaker spoke the Urdu letter pay (‘پ’).

Each video clip in .mp4 format is converted into individual video frames in .jpg format, as shown in Fig. 2. These frames are saved in a folder named identically to the .mp4 video clip file. These image files are then used for the next preprocessing step.

**Fig. 3:**
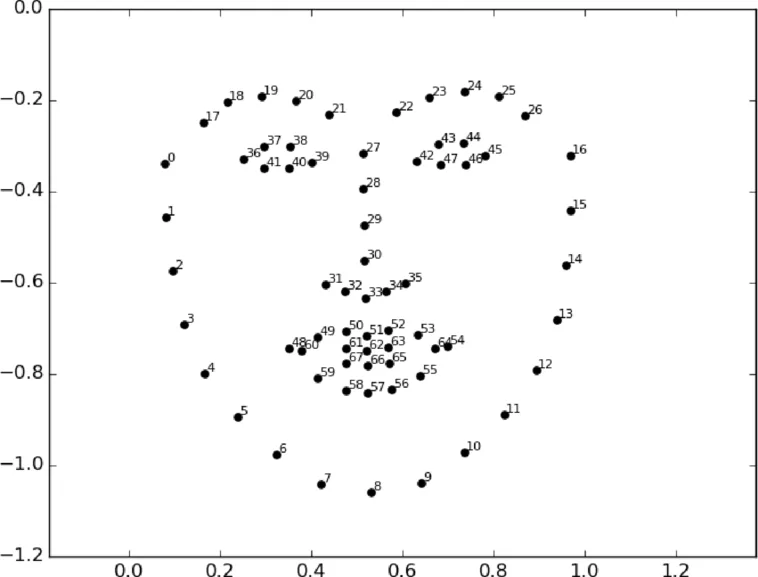
68 standard face land mark used by dlib python library.

The Fig. 2 contains the images from volunteer students for which informed consent to publish the images was obtained as presented in the dataset section.

### Lips extraction

**Fig. 4:**
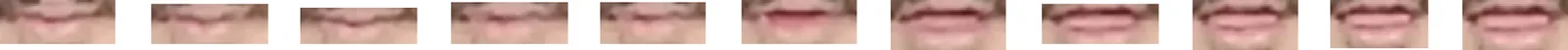
Original lips section of the frames, extracted using python dlib library.

The dlib library is employed to extract the lip region from video frame images. It utilizes 68 spatial landmarks for frontal face detection, as illustrated in Fig. 3. Specifically, landmarks 48 to 67 are dedicated to detecting the mouth and lips. Thus, these 20 landmarks out of the 68 are used to isolate the lip/mouth section from each frame image, as shown in Fig. 4.

### Lips enhancement

**Fig. 5:**
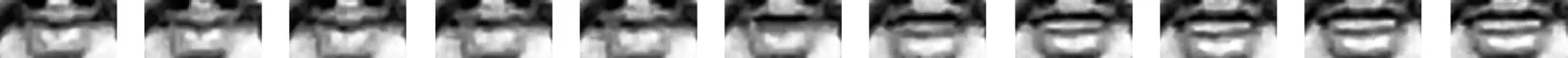
Enhanced and resized (160 × 80) gray scale lip sections.

To enhance the quality of the lip region, the following steps are undertaken: (i) The extracted lip section of each frame is converted from RGB to grayscale, reducing the processing cost per frame for the region of interest (ROI). (ii) Unlike the LipNet model, which requires the speaker to be at a fixed distance from the camera, which is a less flexible and practical approach. The proposed model allows the speaker to be at any distance. This system reads the speaker’s lip movements from the video and predicts the spoken text. The model resizes the lip ROI to 160 × 80, accommodating lips of both small and large sizes by standardizing them to this dimension. (iii) To further enhance the lip region, histogram equalization is applied to the grayscale lip section. This technique balances darker and lighter areas, distributing the shades across the full range of gray tones, as illustrated in Fig. 5. As a result, previously obscured lip details become more visible, enabling the extraction of additional features from the enhanced image.

### DNN models for Urdu alphabets lip reading

To decide on the suitable model for Urdu alphabet lip reading, three models with different structural and classification techniques are trained and tested.

### LipNet-based 2D-CNN model for Urdu alphabets lip reading

**Fig. 6:**
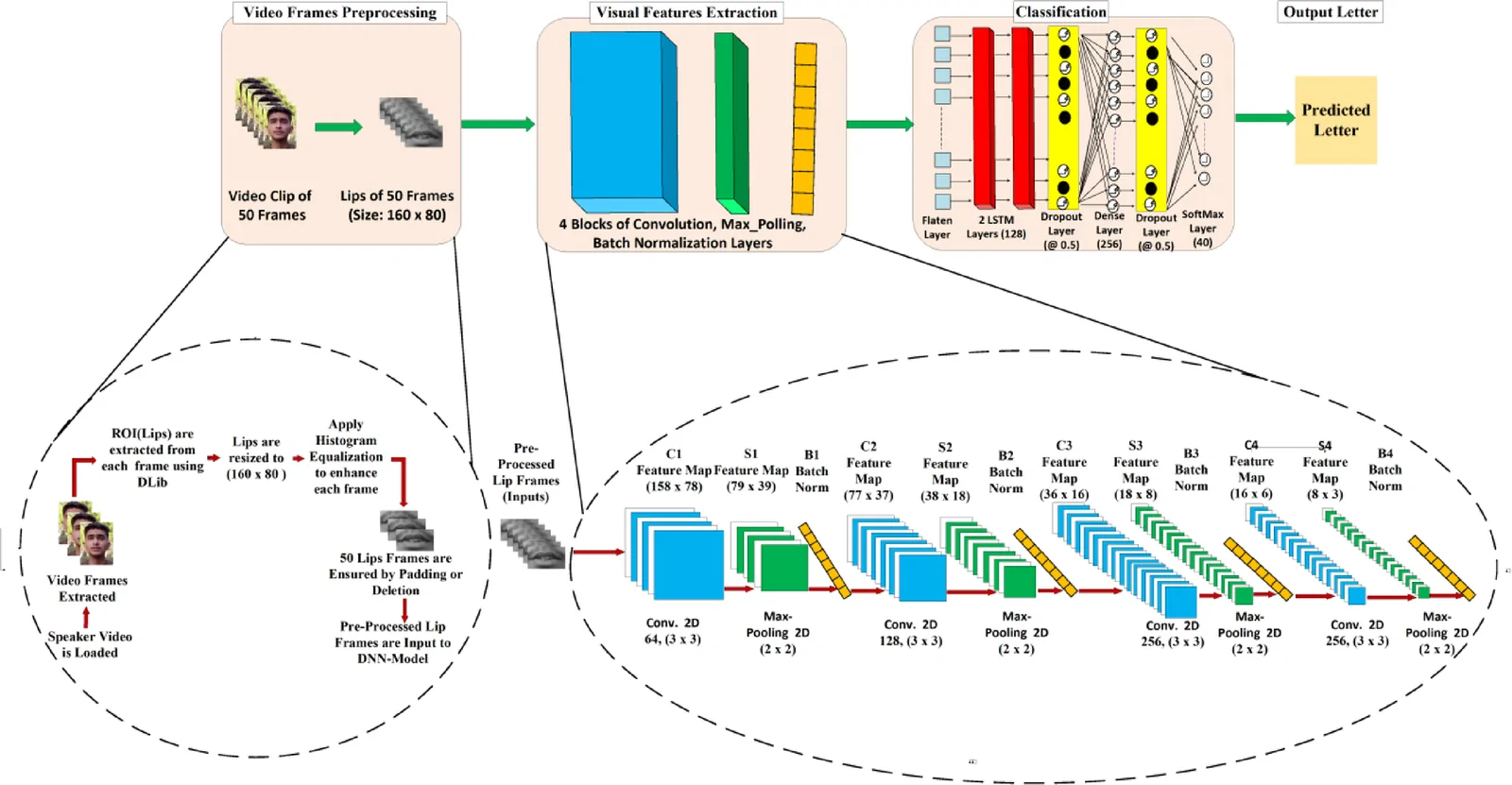
LipNet-based 2D-CNN model for Urdu alphabet lip reading.

The first deep neural network (DNN) model is a 2D-CNN architecture. It comprises four successive two-dimensional convolutional and max-pooling layers, with variations in the convolutional kernel as depicted in Fig. 6.

The model’s layer-wise breakdown is as follows:

The initial layer in the 2D-CNN model is the input layer. The model takes a 5D tensor as input, structured as (BatchSize, FramesPerVideo, height, width, channels). The numeric representation of one batch is determined using Eq. (1) before being fed into the subsequent layer.

1 $$\:\mathrm{X}\in\:\:{\mathrm{R}}^{\mathrm{B}\mathrm{a}\mathrm{t}\mathrm{c}\mathrm{h}\mathrm{S}\mathrm{i}\mathrm{z}\mathrm{e}\:\times\:\:\mathrm{F}\mathrm{r}\mathrm{a}\mathrm{m}\mathrm{e}\mathrm{s}\mathrm{P}\mathrm{e}\mathrm{r}\mathrm{V}\mathrm{i}\mathrm{d}\mathrm{e}\mathrm{o}\:\times\:\:\mathrm{h}\mathrm{e}\mathrm{i}\mathrm{g}\mathrm{h}\mathrm{t}\:\times\:\:\mathrm{w}\mathrm{i}\mathrm{d}\mathrm{t}\mathrm{h}\:\times\:3\:}$$

Following the input layer, the model incorporates four sets of time-distributed layers with 64, 128, 256, and 256 kernels, respectively. Each set consists of three layers: Convolution-2D, MaxPooling-2D, and Batch Normalization. The convolutional layers utilize filters (kernels) to extract spatial features from the input tensor. The 2D convolution operation, employing a (3 × 3) filter, processes each frame independently along the height and width dimensions. This operation is computed using Eq. (2).

2 $$\:\:{{\mathrm{y}}_{\mathrm{i}.\mathrm{j}}^{\left(\mathrm{k}\right)}\:=\:\mathrm{b}}^{\left(\mathrm{k}\right)}\:+\sum\:_{\mathrm{m}=0}^{\mathrm{h}-1}\sum\:_{\mathrm{n}=0}^{\mathrm{w}-1}\sum\:_{\mathrm{l}=0}^{\mathrm{c}-1}{\mathrm{W}}_{\mathrm{m},\mathrm{n},\mathrm{l}}^{\left(\mathrm{k}\right)}\:.\:\:{\mathrm{x}}_{\left(\mathrm{i}+\mathrm{m}\right)\left(\mathrm{j}+\mathrm{n}\right)\mathrm{l}}$$

Where $\:{\mathrm{y}}_{\mathrm{i},\mathrm{j}}^{\left(\mathrm{k}\right)}$ represents the output at location (i, j) for the k-th feature map, b(k) is the bias term, $\:{\mathrm{W}}_{\mathrm{m}\mathrm{n}\mathrm{l}}^{\left(\mathrm{k}\right)}$ denotes the convolutional filter weights for the k-th feature map, $\:{\mathrm{x}}_{\left(\mathrm{i}+\mathrm{m}\right)\left(\mathrm{j}+\mathrm{n}\right)\mathrm{l}}$ is the input value at location (i + m, j + n) and depth l.

The activation function ReLU introduces non-linearity into the DNN, helping to mitigate the vanishing gradient problem that can arise during training. It enables the model to learn more complex relationships in video data. The ReLU function retains positive inputs and sets negative ones to zero, as expressed in Eq. (3).

3 $$\:\mathrm{R}\mathrm{e}\mathrm{L}\mathrm{U}\left(\mathrm{z}\right)=\mathrm{max}\left(0,\mathrm{z}\right)$$

Where, z represents the input to the ReLU activation function.

Max-Pooling reduces the spatial dimensions by selecting the highest value within a (2 × 2) sliding window, computed per frame using Eq. (4).

4 $$\:\mathrm{y}\left[\mathrm{i},\mathrm{j}\right]=\mathrm{max}\left\{\mathrm{x}\left[\mathrm{i}+\mathrm{m},\mathrm{j}+\mathrm{n}\right]\right|\mathrm{m},\:\mathrm{n}\:\in\:\mathrm{p}\mathrm{o}\mathrm{l}\mathrm{l}\mathrm{i}\mathrm{n}\mathrm{g}\:\mathrm{w}\mathrm{i}\mathrm{n}\mathrm{d}\mathrm{o}\mathrm{w}\}$$

Where x is the input feature map and y is the pooled output.

Batch Normalization normalizes the output of a layer to achieve zero mean and unit variance, followed by scaling and shifting with trainable parameters. The calculations are performed using Eqs. (5) and (6).

5 $$\:\widehat{{\mathrm{x}}_{\mathrm{i}}}=\:\frac{{\mathrm{x}}_{\mathrm{i}}-\:{{\upmu\:}}_{\mathrm{b}\mathrm{a}\mathrm{t}\mathrm{c}\mathrm{h}}}{\sqrt{{{\upsigma\:}}_{\mathrm{b}\mathrm{a}\mathrm{t}\mathrm{c}\mathrm{h}}^{2}+\:\epsilon}}$$

6 $$\:{\mathrm{y}}_{\mathrm{i}}={\upgamma\:}\widehat{{\mathrm{x}}_{\mathrm{i}}}+\:{\upbeta\:}\:$$

Where $\:{{\upmu\:}}_{\mathrm{b}\mathrm{a}\mathrm{t}\mathrm{c}\mathrm{h}}$ and $\:{{\upsigma\:}}_{\mathrm{b}\mathrm{a}\mathrm{t}\mathrm{c}\mathrm{h}}^{2}$ represent the mean and variance of the batch, respectively, γ is a scaling factor, β is a shift factor, and *γ* is a small constant for numerical stability.

The Flatten layer reshapes the output from the preceding layers—originally structured as (FramesPerVideo, height, width, channels)—into a single vector format, (FramesPerVideo, features), as defined in Eq. (21). This transformed data is then fed into the subsequent fully connected dense layer.

Next, two LSTM layers, each with 128 units, capture temporal dependencies across frames. Their operations are governed by Eqs. (7)–(12).

7 $$\:{\mathrm{f}}_{\mathrm{t}}={\upsigma\:}\left({\mathrm{W}}_{\mathrm{f}}.\:\:\left[{\mathrm{h}}_{\mathrm{t}-1},{\mathrm{x}}_{\mathrm{t}}\right]+{\mathrm{b}}_{\mathrm{f}}\right)$$

8 $$\:{\mathrm{i}}_{\mathrm{t}}={\upsigma\:}\left({\mathrm{W}}_{\mathrm{i}}.\:\:\left[{\mathrm{h}}_{\mathrm{t}-1},{\mathrm{x}}_{\mathrm{t}}\right]+{\mathrm{b}}_{\mathrm{i}}\right)\:$$

9 $$\:\stackrel{\sim}{{\mathrm{C}}_{\mathrm{t}}}=\mathrm{t}\mathrm{a}\mathrm{n}\mathrm{h}\left({\mathrm{W}}_{\mathrm{C}}.\:\:\left[{\mathrm{h}}_{\mathrm{t}-1},{\mathrm{x}}_{\mathrm{t}}\right]+{\mathrm{b}}_{\mathrm{C}}\right)$$

10 $$\:{\mathrm{C}}_{\mathrm{t}}={\mathrm{f}}_{\mathrm{t}}\mathrm{*}\:{\mathrm{C}}_{\mathrm{t}-1}+\:{\mathrm{i}}_{\mathrm{t}}\mathrm{*}\:\:\stackrel{\sim}{{\mathrm{C}}_{\mathrm{t}}}\:$$

11 $$\:{\mathrm{o}}_{\mathrm{t}}={\upsigma\:}\left({\mathrm{W}}_{\mathrm{o}}.\:\:\left[{\mathrm{h}}_{\mathrm{t}-1},{\mathrm{x}}_{\mathrm{t}}\right]+{\mathrm{b}}_{\mathrm{o}}\right)$$

12 $$\:{\mathrm{h}}_{\mathrm{t}}={\mathrm{o}}_{\mathrm{t}}\mathrm{*}\mathrm{t}\mathrm{a}\mathrm{n}\mathrm{h}\left({\mathrm{C}}_{\mathrm{t}}\right)$$

Where,$\:\:{\mathrm{f}}_{\mathrm{t}}$ is the forget gate, $\:{\mathrm{i}}_{\mathrm{t}}$ is the input gate, $\:{\mathrm{o}}_{\mathrm{t}}$ is the output gate, $\:{\mathrm{C}}_{\mathrm{t}}$ is the cell state, $\:{\mathrm{h}}_{\mathrm{t}}$ is the hidden state, $\:{\mathrm{W}}_{\mathrm{f}}\:,\:{\mathrm{W}}_{\mathrm{i}}.\:{\mathrm{W}}_{\mathrm{C}},\:{\mathrm{W}}_{\mathrm{o}}$ are the weight matrices, and $\:{\mathrm{b}}_{\mathrm{f}},\:{\mathrm{b}}_{\mathrm{i}\:}{,\mathrm{b}}_{\mathrm{C}},\:{\mathrm{b}}_{\mathrm{o}}$ are the bias terms, σ is the sigmoid function, and tanh is the hyperbolic tangent function.

To prevent overfitting, a dropout layer randomly deactivates 50% of the neurons during training. The dropout mechanism is formulated in Eq. (13).

13 $$\:{\widehat{\mathrm{x}}}_{\mathrm{i}}=\:\frac{{\mathrm{x}}_{\mathrm{i}}\:.\:\:{\mathrm{r}}_{\mathrm{i}}}{1-\mathrm{p}}$$

Where, $\:{\mathrm{r}}_{\mathrm{i}}$ is a binary mask that retains $\:{\mathrm{x}}_{\mathrm{i}}$ when set to 1, otherwise dropping it (holding 0), and p represents the dropout probability.

The dense (fully connected) layer applies a linear transformation followed by ReLU activation. The computations are performed using Eq. (14).

14 $$\:\mathrm{y}=\:\mathrm{R}\mathrm{e}\mathrm{L}\mathrm{U}\left(\mathrm{W}.\mathrm{x}+\mathrm{b}\right)$$

Where x is the input feature vector, W is the weight matrix, b is the bias term, and ReLU is the activation function.

For classification, the softmax layer generates a probability distribution across the output classes, calculated using Eq. (15).

15 $$\:\mathrm{s}\mathrm{o}\mathrm{f}\mathrm{t}\mathrm{m}\mathrm{a}\mathrm{x}\left({\mathrm{z}}_{\mathrm{i}}\right)=\:\frac{{\mathrm{e}}^{{\mathrm{z}}_{\mathrm{i}}}}{{\sum\:}_{\mathrm{j}=1}^{\mathrm{n}}{\:\:\mathrm{e}}^{{\mathrm{z}}_{\mathrm{j}}}}$$

Where, z_i_ represents the logits (raw outputs) for each class, and the denominator $\:{\sum\:}_{\mathrm{j}=1}^{\mathrm{n}}{\:\:\mathrm{e}}^{{\mathrm{z}}_{\mathrm{j}}}$ normalizes the sum of exponentials for all logits.

To compute the loss between predicted and actual probability distributions, cross-entropy loss is applied using Eq. (16).

16 $$\:\mathrm{L}\left(\mathrm{y},\:\widehat{\mathrm{y}}\right)=-\sum\:_{\mathrm{i}=1}^{\mathrm{n}}{\mathrm{y}}_{\mathrm{i}}\mathrm{log}\left(\widehat{{\mathrm{y}}_{\mathrm{i}}}\right)$$

Where y is the true label (one-hot encoded), and$\:\:\widehat{{\mathbf{y}}_{\mathbf{i}}}$ represents the predicted probability for class i.

### Hybrid 2D_3D-CNN model for Urdu alphabets lip reading

**Fig. 7:**
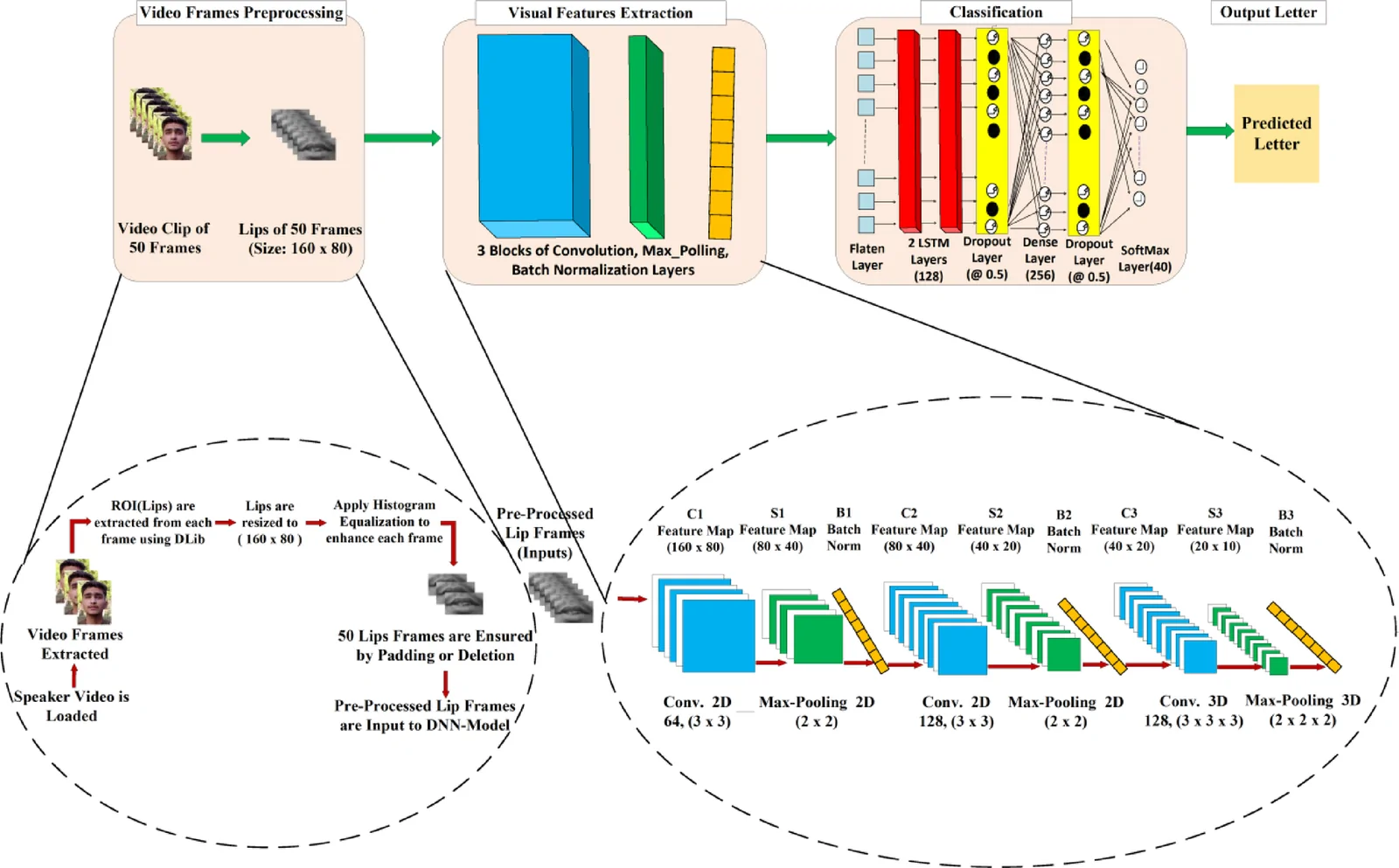
Hybrid 2D_3D-CNN model for Urdu alphabet lip reading.

The second Deep Neural Network (DNN) model is a hybrid architecture that integrates both two-dimensional and three-dimensional convolutional and max-pooling layers, as illustrated in Fig. 7.

The layer-wise structure of the model is described as follows:

The first layer of the Urdu alphabet lip-reading model is the input layer. The model receives input in the form of a 5D tensor with the shape (BatchSize, FramesPerVideo, Height, Width, Channels). The shape of a single batch is numerically transformed using Eq. (1) before being passed to the second layer.

Following the input layer, the model contains two groups of time-distributed layers with 64 and 128 kernels, respectively. Each group consists of three layers: Convolution-2D, MaxPooling-2D, and Batch Normalization. The convolutional layers extract spatial features by applying filters (kernels) to the input tensor. The 2D convolutional layer, with a filter size of (3 × 3), processes frames independently along the height and width dimensions. The convolutional layers perform computations on each frame using Eq. (17).

17 $$\:\left(\mathrm{x}\mathrm{*}\mathrm{w}\right)\left[\mathrm{i},\mathrm{j}\right]=\:\sum\:_{\mathrm{m}}\sum\:_{\mathrm{n}}\mathrm{x}\left[\mathrm{i}+\mathrm{m},\:\:\mathrm{j}+\mathrm{n}\right]\mathrm{w}\left[\mathrm{m},\mathrm{n}\right]+\mathrm{b}$$

Where x represents the input feature map (spatial dimensions), w is the convolution filter (kernel), b is the bias term, and i, j are spatial indices across the height and width of the input.

Max-Pooling reduces spatial dimensions by selecting the maximum value within a sliding window of size (2 × 2). The computations in this layer are performed per frame using Eq. (4).

Batch Normalization normalizes the layer output to achieve zero mean and unit variance, followed by scaling and shifting with learnable parameters. The normalization process follows Eqs. (18) and (19).

18 $$\:\widehat{\mathrm{x}}=\:\frac{\mathrm{x}-\:{\upmu\:}}{\sqrt{{{\upsigma\:}}^{2}+\:\epsilon}}$$

19 $$\:\mathrm{y}={\upgamma\:}\widehat{\mathrm{x}}+\:{\upbeta\:}$$

Where µ is the mean of the batch, σ2 is the variance, γ is a scaling factor, β is a shift factor, and $\:\epsilon$ is a small constant for numerical stability.

Next, the model incorporates a group of 3D-Convolutional, 3D-MaxPooling, and Batch Normalization layers. The 3D convolutional layer applies a 3D filter of size (3 × 3 × 3) across both the temporal dimension (frames) and spatial dimensions (height and width) to extract spatiotemporal features. The computations for 3D convolution on each frame are given by Eq. (20).

20 $$\:\left(\mathrm{x}\mathrm{*}\mathrm{w}\right)\left[\mathrm{i},\:\mathrm{j},\:\mathrm{k}\right]=\:\sum\:_{\mathrm{m}}\sum\:_{\mathrm{m}}\sum\:_{\mathrm{p}}\mathrm{x}\left[\mathrm{i}+\mathrm{m},\:\mathrm{j}+\mathrm{n},\:\mathrm{k}+\mathrm{p}\right]\mathrm{w}\left[\mathrm{m},\mathrm{n},\:\mathrm{p}\right]+\mathrm{b}$$

Where x represents the input tensor (time, height, width), w is the 3D filter (kernel), and i, j,k are indices across the temporal and spatial dimensions.

3D Max-Pooling reduces frame size by down sampling across the time, height, and width dimensions using a (2 × 2 × 2) pooling window, as described in Eq. . (21).

21 $$\:\mathrm{y}\left[\mathrm{i},\mathrm{j},\mathrm{k}\right]=\mathrm{max}\left\{\mathrm{x}\left[\mathrm{i}+\mathrm{m},\mathrm{j}+\mathrm{n},\:\mathrm{k}+\mathrm{p}\right]\right|\mathrm{m},\:\mathrm{n},\:\mathrm{p}\:\in\:\mathrm{p}\mathrm{o}\mathrm{l}\mathrm{l}\mathrm{i}\mathrm{n}\mathrm{g}\:\mathrm{w}\mathrm{i}\mathrm{n}\mathrm{d}\mathrm{o}\mathrm{w}\}$$

Where x is the input tensor, and y is the pooled output.

Similarly, Batch Normalization normalizes the output and regularizes the training process.

The next layer is the Flatten layer, which reshapes the tensor from (FramesPerVideo, Height, Width, Channels) to (FramesPerVideo, Features) by collapsing the spatial dimensions. The reshaped tensor is computed using Eq. (22).

22 $$\:{\mathrm{X}}_{\mathrm{n}\mathrm{e}\mathrm{w}}=\:\mathrm{R}\mathrm{e}\mathrm{s}\mathrm{h}\mathrm{a}\mathrm{p}\mathrm{e}\left(\mathrm{X}\right)\:$$

Where the tensor is reformatted while maintaining the same number of elements.

Following this, the model incorporates two Long Short-Term Memory (LSTM) layers for temporal modeling. Each LSTM unit maintains a hidden state ht ​ and a cell state ct​, updated with the input xt at each time step. The operations for these layers are governed by Eqs. (7)–(12).

Dense layers perform a linear transformation on the input, followed by a non-linear activation function. The computations for the Dense layer are given by Eq. (14).

To prevent overfitting, the Dropout layer randomly sets a fraction p of the input units to zero during training. The Dropout layer computations are performed by Eq. (23).

23 $$\:\mathrm{y}=\left\{\begin{array}{c}0,\:\:\:\:\:\:with\:probability\:p\:\:\:\:\:\:\:\\\:x,\:\:\:\:\:\:with\:probability\:1-p\end{array}\:\right.$$

Where p represents the dropout rate, and x is the input to the layer.

The Softmax layer converts output logits into a probability distribution over the classes, computed using Eq. (15).

Finally, the model uses the Categorical Cross-Entropy loss function to measure the difference between the predicted probability distribution and the true distribution, as described in Eq. (16).

### 3D-CNN model for Urdu alphabets lip reading with mixed precision

**Fig. 8:**
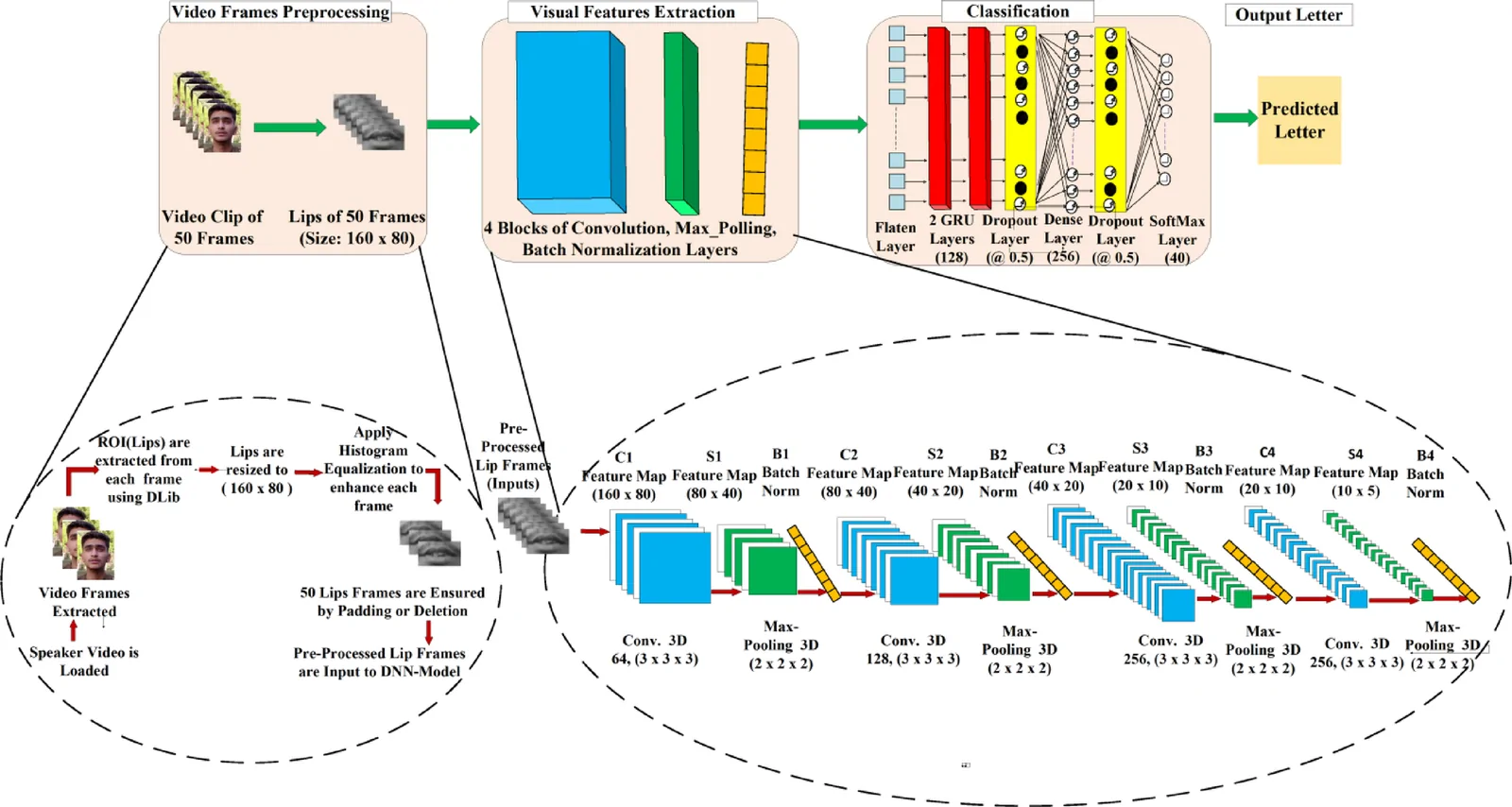
3D CNN model for Urdu alphabet lip reading with mixed precision.

The third deep neural network (DNN) model is based on a 3D Convolutional Neural Network (3D CNN). It comprises four sequential three-dimensional convolutional and max-pooling layers, with variations in convolution kernel sizes, as depicted in Fig. 8.

The model architecture is structured as follows:

The first layer of the 3D CNN model is the input layer. The model receives input in the form of a 5D tensor with dimensions (BatchSize, FramesPerVideo, Height, Width, Channels). The shape of a single batch is transformed into numeric form using Eq. (1) before being passed to the subsequent layer.

Following the input layer, the model consists of four sets of 3D layers with 64, 128, 256, and 256 kernels, respectively. Each set includes three layers: a 3D Convolutional layer, a 3D Max-Pooling layer, and a Batch Normalization layer. The 3D convolutional layers apply a filter (kernel) ‘W’ of shape (*F*_*t*_, *F*_*h*_, *F*_*w*_, *C*, *K*) (filter time, filter height, filter width, input channels, output channels) to the input tensor ‘I’ with shape (T, H, W, C) (time, height, width, channels) to extract spatial features. The convolution operation is executed on a single frame using Eq. (24).

24 $$\:O(t,h,w,k)\:=\:\sum\:_{c=1}^{C}\sum\:_{i=1}^{{F}_{t}}\sum\:_{j=1}^{{F}_{h}}\sum\:_{l=1}^{{F}_{w}}W\left(i,j,l,c,k\right).\:I\left(t+i,h+j,w+l,c\right)\:$$

Where O represents the output cube, t, h, and w correspond to time, height, and width indices, and k is the output channel index.

A ReLU activation function is applied using Eq. (3) to introduce non-linearity into the DNN.

The 3D Max-Pooling layer performs down-sampling by selecting the maximum value within a sliding cube of size (2 × 2 × 2). The operation on a single frame is computed using Eq.. (25).

25 $$\:O\left(t,h,w,c\right)={max}_{(i,j,l)\in\:P}\:I\left(t+i,h+j,w+l,c\right)$$

Where ‘I’ represents the input cube, and P denotes the pooling region of dimensions $\:\left({P}_{t},{P}_{h},{P}_{w}\right)$ for time, height, and width.

The Batch Normalization layer normalizes the output of the previous layer to achieve zero mean and unit variance, then scales and shifts it using learnable parameters. The normalization process follows Eq. (5) and Eq. (6).

The Flatten layer transforms the spatial dimensions (height, width, and channels) of the previous layer’s output into a one-dimensional vector while preserving the time dimension (frames). This transformation is performed using Eq. (22).

The gated recurrent unit (GRU) layer processes the flattened tensor by capturing temporal dependencies. For each time step t, the reset gate $\:{\mathrm{r}}_{\mathrm{t}}$, update gate $\:{\mathrm{z}}_{\mathrm{t}}$, candidate hidden state $\:\stackrel{\sim}{{h}_{t}}$, and final hidden state $\:{\mathrm{h}}_{\mathrm{t}}$ are computed using Eqs. (26)–(29).

26 $$\:{r}_{t}\:=\:\sigma\:\left({W}_{r}\:.\:\:{x}_{t}+\:{U}_{r}\:.\:\:{h}_{t-1}+\:{b}_{r}\right)$$

27 $$\:{z}_{t}\:=\:\sigma\:\left({W}_{z}\:.\:\:{x}_{t}+\:{U}_{z}\:.\:\:{h}_{t-1}+\:{b}_{z}\right)$$

28 $$\:\stackrel{\sim}{{h}_{t}}\:=\:tanh\left({W}_{h}\:.\:\:{x}_{t}+\:{U}_{h}\:.\:\left(\:{{r}_{t}*\:h}_{t-1}\right)+\:{b}_{h}\right)$$

29 $$\:{h}_{t}\:=\:\left(1-{z}_{t}\right)*{h}_{t-1}+\:{z}_{t}\:*\stackrel{\sim}{{h}_{t}}\:$$

Where *σ* is the sigmoid activation function, * denotes element-wise multiplication, *W*_*r*_, *W*_*z*_, *W*_*h*_, *U*_*r*_, *U*_*z*_, *U*_*h*_ are the weight matrices, and *b*_*r*_, *b*_*z*_, *b*_*h*_ are biases.

Next, the dropout layer converts all the negative values of the tensor to zero to prevent overfitting in input data using Eq. (13).

The Dense (fully connected) layer follows, computing a weighted sum of inputs from the previous layer, followed by the application of the ReLU activation function using Eqs. (3), (14).

The Softmax layer then applies the softmax function, as given in Eq. (15), to generate a probability distribution over the output classes of the DNN model.

Finally, the model is compiled using the cross-entropy loss function, which is calculated using Eq. (16). This function optimizes the model by adjusting the weights during training, minimizing the error between predicted and actual values.

### Construction of ULRA (Urdu lip reading alphabets) dataset

In this section, a method for alphabet dataset construction, augmented dataset, padding of frames, and dataset classes are described in detail.

### Dataset method

Since the Urdu alphabet dataset is the foundation for Urdu lip reading research, so for the construction of the Urdu alphabet dataset, the following steps were performed. These steps include: (i) Description of the Urdu alphabet dataset vocabulary, (ii) Recording of the Urdu alphabet dataset vocabulary, (iii) Extraction of video clips, and (iv) Preparation of text files. Each step is elaborated in the following sections.

#### Description of Urdu alphabet dataset vocabulary

The Urdu alphabet dataset vocabulary comprises 40 distinct Urdu letters, ranging from alif “ا” to barri ye “ے”. Each letter is assigned a unique code, denoted as “at”, for annotation and labeling purposes. For instance, “at001.mp4” to “at040.mp4” represent video files, while “at001.txt” to “at040.txt” correspond to the respective text files for each letter.

#### Recording of Urdu alphabet dataset vocabulary

The dataset consists of 40 classes recorded by 15 native Urdu speakers. All dataset construction methods adhered to ethical guidelines and regulations. All experimental protocols were approved by a Hazara University, Mansehra Pakistan and Ethics and ASRB licensing committee. Moreover, all the methods were performed in accordance with relevant guidelines and regulations of nature journal given at web location: https://www.nature.com/srep/journal-policies/editorial-policies#experimental-subjects. The participants were volunteer university students, and written consent was obtained from each individual. Considering their academic schedules, each speaker participated in the recording process on their available day. The dataset recording spanned 15 working days, commencing on October 10, 2023, and concluding on October 31, 2023.

Each speaker recorded all 40 classes twice, once in a controlled indoor environment and once in an uncontrolled outdoor setting, resulting in two separate video recordings per letter. Every letter was recorded 30 times in a frontal view under both conditions. Mobile phones were used for recording, with cameras set to 1080p resolution at 30 FPS. The camera-to-speaker distance was maintained at approximately 1.5 m.

#### Extraction of video clips

Extracting individual clips from full-length recordings was a meticulous and highly technical phase. To ensure precision, 10 experienced editors, proficient in CapCut video editing tools, were selected. The editors were instructed on the importance of dataset accuracy in research. They were required to carefully segment the long recordings into 40 distinct video clips, ensuring that: Overlapping of clips was strictly avoided. Each clip was correctly labeled with an appropriate filename. The final dataset maintained high accuracy by precisely cropped clips with proper annotations.

#### Text file preparation

Each extracted video clip was accompanied by a corresponding text file, created and edited in Notepad. These files contain the ground truth for the spoken words in the video. They were saved using the UTF-8 encoding format with a .txt extension and named identically to their respective video files.

### Augmented dataset

**Fig. 9:**
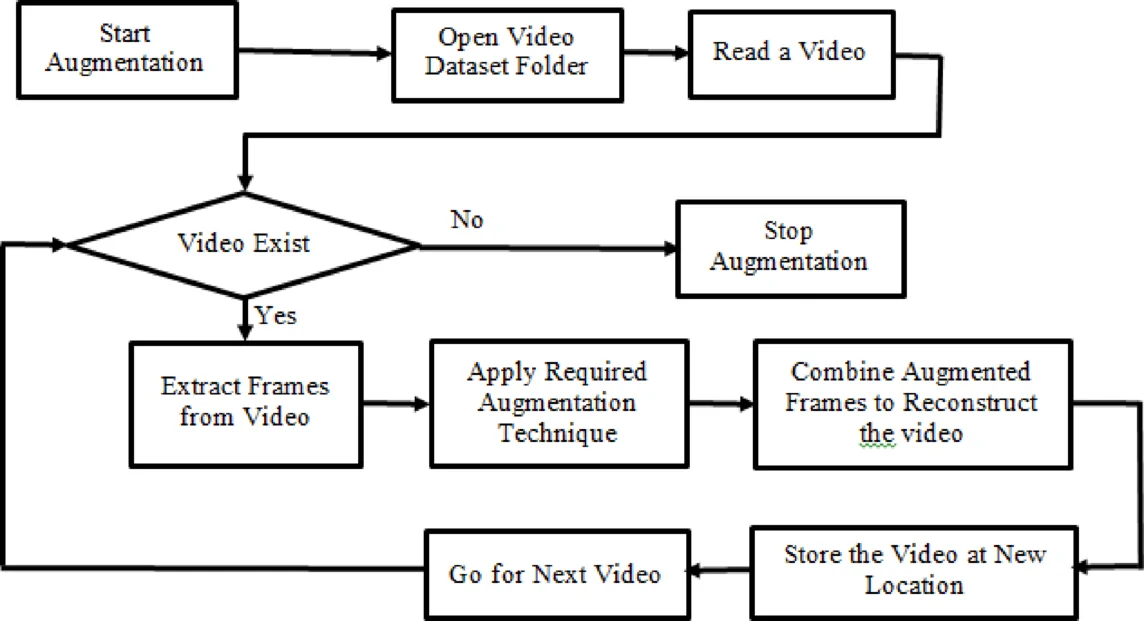
Flowchart diagram for generating the part of the augmented dataset.

To enhance the model’s generalization capabilities, video augmentation techniques were applied to the dataset. This not only mitigates overfitting issues but also improves the learning process during training. These techniques effectively increase dataset size and introduce variations in the data. The augmentation techniques used in this research includes: translation, horizontal flipping, rotation (+ 10 degree), and rotation (-10 degree) from spatial augmentation, color jittering i.e., color adjustment of brightness, contrast, saturation, and hue from color augmentation, and blurring from noise and blurring techniques. By employing these augmentation strategies, the dataset is expanded to six times its original size. The flow diagram to perform the data augmentation on videos is shown in Fig. 9.

**Fig. 10:**
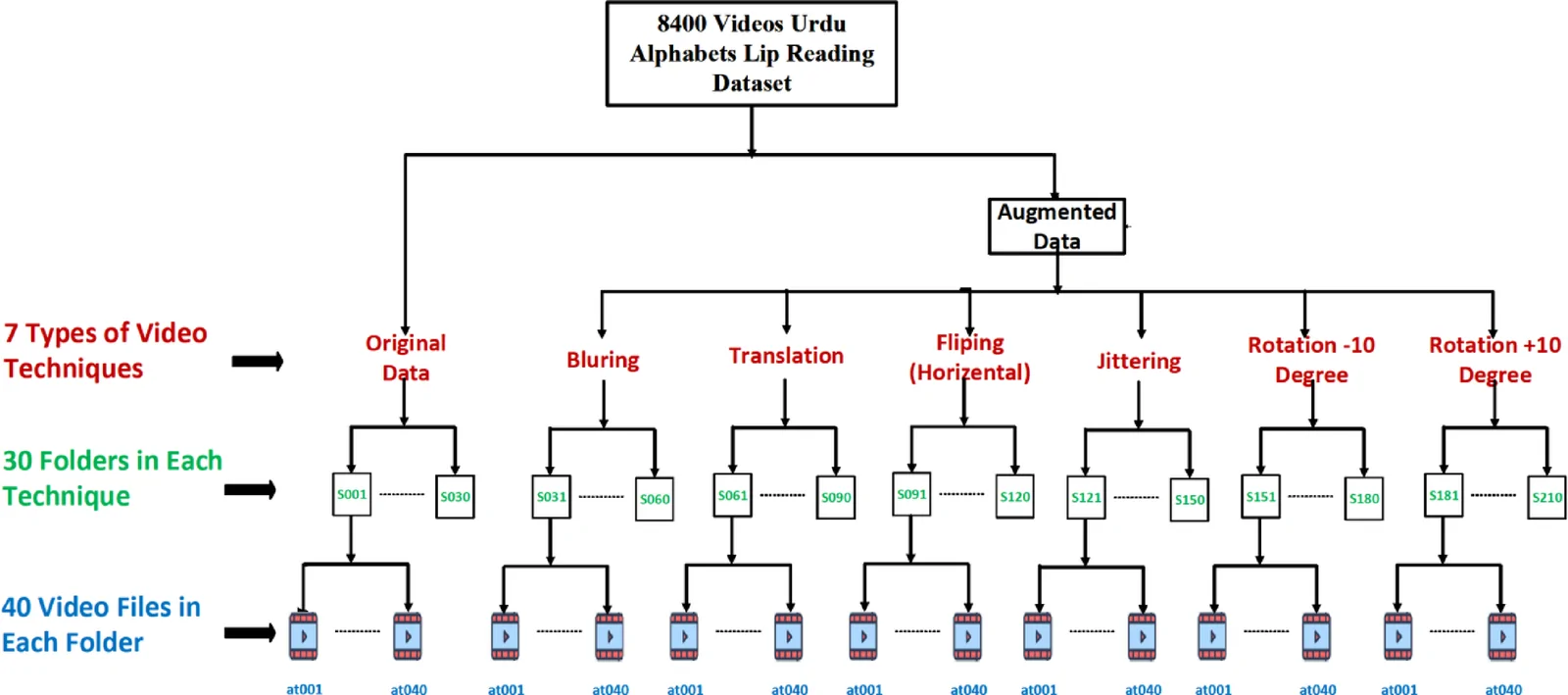
Tree diagram of ULRA dataset.

The impact of various augmentation techniques on the dataset is summarized in Table 1. The dataset is structured into seven groups, with video clips organized accordingly. Column 3 of Table 1 details the folder ranges where specific augmented data is stored. Figure 10 illustrates the complete organization of the Urdu Lip Reading Alphabet (ULRA) dataset. The dataset comprises a total of 210 folders, with each group containing 30 folders. Each folder includes 40 .mp4 video clips, corresponding to Urdu alphabet recordings.

Thus, the contribution of each augmentation technique results in **1**,**200 additional video clips** per group. The total count of video clips in the dataset is: 1200 × 7 = 8400 video clips. Each batch of **40 videos** is stored in one of the **210 dataset folders**, labeled as: **S001**,** S002**,** S003**,** …**,** S210**. The Urdu Lip Reading Alphabet dataset is uploaded on GitHub platform and is publically available at URL: https://github.com/umair1977/ULRA/blob/main/dataset%20link.

**Table 1:** Order in which different augmentation techniques data is stored in the dataset.

G.no	Actual/augmented technique	Folders range in dataset	Sample video frames of actual/augmented videos					
1	Actual data frames	S001 To S030						
2	Blur data frames	S031 To S060						
3	Translation frames	S061 To S090						
4	Flip (horizontal)	S091 To S120						
5	Jittering data frames	S121 To S150						
6	Rotate -10^o^	S151 To S180						
7	Rotate +10^o^	S181 To S210						

### Padding frame

It’s worth noting that the lengths of video clips representing the pronunciation of Urdu alphabets are not uniform; rather, they vary due to differences in pronunciation speed, with some alphabets being articulated more quickly or slowly. Additionally, this length can differ because of accent-related delays by certain speakers when pronouncing the same alphabet. Deep neural networks, however, require video clips of equal length, making this variation challenging. To address this, a padding technique is used. Padding involves duplicating the original frames of the shorter video clips in a way that preserves the natural timing and accent of the speaker’s delivery. Extra frames are added by duplicating each original frame to its next index position, continuing until the clip reaches the required frame count. This padding approach offers several advantages:

i) The system avoids dealing with unnecessary zero-value padded frames.
ii) The spatio-temporal sequence of frames remains intact, thereby preserving the original order of spatio-temporal features.

### ULRA dataset classes

ULRA Dataset consists of forty classes as shown in Table 2. The first and fourth column of the table shows Urdu alphabet symbols, In the second and fifth columns Urdu pronunciation of the alphabet is given, third and sixth column shows roman Urdu Pronunciation.

**Table 2:** Urdu language alphabet pronunciation.

Alphabet	Urdu pronunciation	Roman Urdu pronunciation	Alphabet	Urdu pronunciation	Roman Urdu pronunciation
ا	الف	Alif	ص	صاد	Saad
آ	الف مد آ	Alif mad aa	ض	ضاد	Zaad
ب	بے	Bay	ط	طؤے	Toye
پ	پے	Pay	ظ	ظؤے	Zoye
ت	تے	Tey	ع	عین	Ain
ٹ	ٹے	Tay	غ	غین	Ghain
ث	سے	Say	ف	فے	Fay
ج	جیم	Jeem	ق	قاف	Kaaf
چ	چیم	Cheem	ک	کیاف	Kiyaf
ح	بڑی ھیے	Barri hey	گ	گاف	Gaaf
خ	خے	Khey	ل	لام	Laam
د	دال	Dal	م	میم	Meem
ڈ	ڈال	Daal	ن	نون	Noon
ذ	ذال	Zaal	ں	نون غنہ	Noon ghunnah
ر	رے	Ray	و	واؤ	Wao
ڑ	ڑے	Aray	ہ	چھوٹی ھیے	Choti hey
ز	زے	Zey	ھ	دو چشمی ھیے	Dow chashmi hey
ژ	ژوے	Xooye	ء	ہمزہ	Hamza
س	سین	Seen	ی	چھوٹی یہ	Choti ye
ش	شین	Sheen	ے	بڑی یہ	Barri ye

## Results and discussion

In this section, the behavior of three models during their training and validation process is discussed under different parameters setting. Moreover, the testing results on test data of these models are also found to their accurate in different conditions like seen and unseen data, control, and un-control environment effects on model accuracy. Furthermore, the letter level analysis through confusion matrices of all three models is also presented.

### Training and validation analysis of all three models

Table 3, shows the training and validation accuracy, and loss factors of three DNN models. The following discussion presents the data trends shown in these graphs, during the training process of these models.

### Training and validation analysis of 2D-CNN model

The first graph of Table 3 shows the data trends during the training and validation process of the 2D-CNN model. The training accuracy improves gradually from 2.8% to 96.6% up to epoch 60, showing that the model can increasingly predict the training data correctly. On the other hand, the validation accuracy also increases up to epoch 60 but is a little bit slow. Moreover, the fluctuations are observed during validation for most of the epochs and cause to increase in the gap between the two accuracy lines. The gap between training and validation accuracy shows overfitting. This implies that the model was performing well on training data but struggling with validation data during the training process. The model was evaluated with different batch size like 16, and 28. The model shows better accuracy on batch size 28 as compare to batch size 16.

The second graph of Table 3 shows the data trends during training and validation loss of the 2D-CNN model. The training loss starts from 3.7 and ends to 0.1 by gradually decreasing its level. The data trends interpret that the model learned effectively from the training data. In contrast, the validation loss also decreases consistently showing that the model is generalizing itself but after 15 epochs the fluctuation in validation loss started, and a gap was produced between training and validation loss, which indicates a little bit overfitting issue.

According to graphs analysis, the expected accuracy of the 2D-CNN model is 96% and 82% on seen and unseen data respectively, while overfitting issues demand a more increase in training data.

### Training and validation analysis of hybrid 2D_3D-CNN model

The third graph of Table 3, presents the 2D_3D-CNN model’s training and validation accuracy. It is observed that training accuracy steadily increases and reaches 93% at epoch 74. This indicates that the model is capturing important patterns and will generalize well on training data. On the other hand validation accuracy rises side by side with training accuracy but after 20 epochs, the fluctuation in validation accuracy started. At the end of the training process, the validation accuracy rises to 78%. The gap occurs, as the training accuracy increases and validation accuracy decreases, which causes the overfitting issue of the model. The model was evaluated with different batch size like 08, 12 and 14. The model shows better accuracy on batch size 14 as compare to batch sizes 08, and 14.

The fourth graph of Table 3 shows the training loss gradually decreases, which shows that the model is successfully learning from the training data. The validation loss also decreases as the epochs increase. The fluctuation in loss after 15 epochs occurs, and causes in the increase in gap between training and validation loss. The small loss in training and more loss in validation fall the model to overfitting problem. This model also demands more training data to generalize itself for unseen data.

### Training and validation analysis of 3D-CNN model

The Training and validation performance of the 3D-CNN model is shown in the fifth graph of Table 3. The training accuracy improves steadily to 69% at epoch 55, and the validation accuracy rises from around 3% to approximately 51%, indicating that the model is indeed learning but with some variability. The model was evaluated with different batch size like 06, 08 and 12. The model shows better accuracy on batch size 08 as compare to batch sizes 06, and 12.

**Table 3:** Training and validation accuracy and loss graph of three DNN models.

Trained model name	Training/validation accuracy	Training/validation loss
2D-CNN model		
2D_3D-CNN model		
3D-CNN model		

The sixth graph of Table 3, indicates that the training loss decreases consistently from 3.77 to approximately 0.78, this means that the model is learning from the training data over time. The validation loss also trends downward overall, with fluctuations that might suggest difficulties in generalization.

The graphs given in Table 3 show the training and validation, accuracy, and loss of models. In this graph, we can analyze the overfitting and underfitting behaviors of the models. It is observed, that almost all the models were falling in the overfitting problems. Then in this situation, it was necessary to see that although all the models are overfitted, instead which one is less overfitted among these three. So the close analyses of three models with respect to training and validation accuracy, and training and validation loss are presented in Table 4. The training and validation accuracy and loss data of all models during their training process are recorded in such an order that the first reading is recorded at epoch 5 and with an interval of 5, 12 subsequent readings of all models in four required factors are noted. The first, second, third, and fourth graph shows the training accuracy, validation accuracy, training loss, and validation loss of all three models respectively. The graphical data depicts that the 2D-CNN Urdu lip reading model was best in all respects. Next, the testing accuracy of all three models will be plotted by the test data and will be compared accordingly.

**Table 4:** Comparative analysis of three Urdu alphabets lip reading models.

Metric	Training accuracy/loss graphs	Validation accuracy/loss graphs
Accuracy		
Loss		

### Testing results of all three models and their comparative analysis

Three models are trained, evaluated, and tested using the ULRA dataset. Individual results of each model on different sections of datasets are shown in Tables 6, 7, and 8, while the combined comparative analysis of the three models is shown in Tables 9, and 10.

The data of Tables 6, 7, and 8 is compiled and graphs of three models 2D-CNN, Hybrid 2D_3D-CNN, and 3D-CNN shown in Table 5 on seen and unseen data in control and un-control environments are drawn for analyzing and comparing the performance on test data.

**Table 5:** Testing accuracy on seen and unseen data in control and un-control environments.

Data	Control environment	Un-control environment
Seen		
Un-Seen		

In a controlled environment with seen data, the 2D-CNN model achieves a higher accuracy than the other two models, except when tested on actual video data, where the Hybrid model shows a slightly higher accuracy—about 2% more than the 2D-CNN. In this scenario, the Hybrid model also shows a noticeably higher accuracy compared to the 3D-CNN model.

In an uncontrolled environment with seen data, the 2D-CNN model maintains the highest accuracy among the three techniques. For the horizontal flip technique, however, its accuracy is slightly lower, by about 3%, than that of the Hybrid model. The accuracy of both the 2D-CNN and Hybrid models remains similar on actual, blurred, and translated video data, if compared to the other two models, the accuracy of the Hybrid model again outperforms the 3D-CNN.

In contrast, when these three models are evaluated on unseen data in a controlled environment, it is noted that the accuracy of the 2D-CNN model matches the Hybrid model for actual videos, while for the remaining data; the 2D-CNN consistently outperforms the Hybrid model. However, in this scenario, the Hybrid model shows relatively higher accuracy than the 3D-CNN model.

In an uncontrolled environment with unseen data, including blurred, jittered, and rotated (+ 10°) videos, the accuracy of the 2D-CNN and Hybrid models is equal, while for other techniques video’s data, the 2D-CNN maintains a higher accuracy. However, the Hybrid model consistently achieves relatively higher accuracy than the 3D-CNN model, except in the case of horizontal flips, where the 3D-CNN outperforms it by approximately 5%.

**Table 6:** Results of LipNet-based 2D-CNN model for Urdu alphabet lip reading.

S.#	Dataset type	Control environment						Un-control environment					
		Seen data			Un-seen data			Seen data			Un-seen data		
		Correct videos	Incorrect videos	Accuracy (%)	Correct videos	Incorrect videos	Accuracy (%)	Correct videos	Incorrect videos	Accuracy (%)	Correct videos	Incorrect videos	Accuracy (%)
1	Actual videos	37	3	92.50	33	7	82.5	39	1	97,5	36	4	90.00
2	Blur videos	40	0	100.0	38	2	95.00	36	4	90.00	39	1	97.50
3	Translate videos	38	2	95.00	40	0	100.0	38	2	95.00	37	3	92.50
4	Horizontal flipped	38	2	95.00	32	8	80.00	38	2	95.00	15	25	37.50
5	Jittered video	40	0	100.0	38	2	95.00	36	4	90.00	29	11	72.50
6	Rotated –ve 10^o^	38	2	95,00	36	4	90.00	39	1	97,5	27	13	67.50
7	Rotated +ve 10^o^	40	0	100.0	33	7	82.50	39	1	97,5	26	14	65.00
	Average accuracy	271	9	96.79	250	30	89.29	265	15	94,64	209	71	74.64

**Table 7:** Results of hybrid 2D_3D-CNN model for Urdu alphabet lip reading.

S.#	Dataset type	Control environment						Un-control environment					
		Seen data			Un-seen data			Seen data			Un-seen data		
		Correct videos	Incorrect videos	Accuracy (%)	Correct videos	Incorrect videos	Accuracy (%)	Correct videos	Incorrect videos	Accuracy (%)	Correct videos	Incorrect videos	Accuracy (%)
1	Actual videos	38	2	95.0	33	7	82.50	39	1	97.50	30	10	75.00
2	Blur videos	40	0	100.0	36	4	90.00	36	4	90.00	39	1	97.50
3	Translate videos	37	3	92.5	39	1	97.50	38	2	95.00	31	9	77.50
4	Horizontal flipped	34	6	85.0	24	16	60.00	39	1	97.50	4	36	10.00
5	Jittered video	36	4	90.0	32	8	80.00	31	9	77.50	30	10	75.00
6	Rotated –ve 10^o^	37	3	92.5	30	10	75.00	38	2	95.00	23	17	57.50
7	Rotated +ve 10^o^	37	3	92.5	12	28	30.00	38	2	95.00	26	14	65.00
	Average accuracy	259	21	92.50	206	74	73.57	259	21	92.50	183	97	65.36

**Table 8:** Results of 3D-CNN model for URDU Alphabet lip reading with mixed precision.

S.#	Dataset type	Control environment						Un-control environment					
		Seen data			Un-seen data			Seen data			Un-seen data		
		Correct videos	Incorrect videos	Accuracy (%)	Correct videos	Incorrect videos	Accuracy (%)	Correct videos	Incorrect videos	Accuracy (%)	Correct videos	Incorrect videos	Accuracy (%)
1	Actual videos	30	10	75.00	24	16	60.00	34	6	85.00	18	22	45.00
2	Blur videos	37	3	92.50	29	11	72.50	28	12	70.00	32	8	80.00
3	Translate videos	33	7	82.50	30	10	75.00	32	8	80.00	27	13	67.50
4	Horizontal flipped	29	11	72.50	21	19	52.50	32	8	80.00	6	34	15.00
5	Jittered video	31	9	77.50	28	12	70.00	26	14	67.50	21	19	52.50
6	Rotated –ve 10^o^	31	9	77.50	20	20	50.00	27	13	67.50	13	27	32.50
7	Rotated +ve 10^o^	32	8	80.00	5	35	12.50	33	7	82.50	12	28	30.00
	Average accuracy	223	57	79.64	157	123	56.07	212	68	75.71	129	151	46.07

For analyzing the model’s performance, the accuracy results of Tables 6, 7, and 8 are further arranged with respect to control and uncontrolled environment and also with respect to seen and unseen data, in Tables 9 and 10 respectively. Table 9 shows, the overall accuracies of 2D-CNN, hybrid 2D_3D-CNN, and 3D-CNN models 93.02, 83.02, 67.86 in the control environment and 84.64, 78.93, 60.89 in uncontrol environments respectively. These results show that the highest accuracies 93.02 and 84.64 of the 2D-CNN model in control and uncontrol environments make this model the best model among the three.

**Table 9:** Test results of three models in control and un-control environment.

Multiple models	Overall accuracy in control environment			Overall accuracy in un-control environment		
	Seen data	Un-seen data	Overall accuracy	Seen data	Un-seen data	Overall accuracy
LipNet-based 2D-CNN model	96.75	89.29	**93.02**	94.64	74.64	**84.64**
Hybrid 2D_3D- CNN model	92.50	73.54	**83.02**	92.50	65.36	**78.93**
3D-CNN model	79.64	56.07	**67.86**	75.71	46.07	**60.89**

**Table 10:** Test results of three models on seen and un-seen data.

Multiple models	Overall accuracy on seen data			Overall accuracy on un-seen data		
	Control environment	Un-control environment	Overall accuracy	Control environment	Un-control environment	Overall accuracy
LipNet-based 2D-CNN model	96.75	94.64	**95.70**	89.29	74.64	**81.97**
Hybrid 2D_3D-CNN model	92.50	92.50	**92.50**	73.54	65.36	**69.45**
3D-CNN model	79.64	75.71	**77.68**	56.07	46.07	**51.07**

Similarly, Table 10 shows the overall accuracies of 2D-CNN, hybrid 2D_3D-CNN, and 3D-CNN models 95.70, 92.50, 77.68 of seen data and 81.97, 69.45, 51.07 of unseen data respectively. These results show that the highest accuracies 95.70 and 81.97 of the 2D-CNN model for seen and unseen data make this model the best model among the three. After analyzing the testing results from different aspects, it is noted that the 2D-CNN model is the best Urdu alphabet lip reading model.

### Comparative analysis of this research with^38^

Tables 11 and 12 present a comparative evaluation of three Urdu lip-reading models—LipNet-based 2D-CNN, Hybrid 2D–3D CNN, and 3D-CNN—on Urdu digits and Urdu alphabets under uncontrolled environments and unseen data conditions.

**Table 11:** Comparison of Urdu digits and alphabets results of three models on unseen in un-control environment.

Multiple models	Overall accuracy in un-control environment	
	Reference^38^ Overall accuracy on Urdu digits	Overall accuracy on Urdu alphabets
LipNet-based 2D-CNN model	87.15	84.64
Hybrid 2D_3D-CNN model	82.86	78.93
3D-CNN model	75.72	60.89

**Table 12:** comparison of Urdu digits and alphabets results of three models on un-seen data in mixed environment.

Multiple models	Overall accuracy on un-seen data	
	Reference^38^ Overall accuracy on Urdu digits	Overall accuracy on Urdu alphabets
LipNet-based 2D-CNN model	90.72	81.97
Hybrid 2D_3D-CNN model	90.00	69.45
3D-CNN model	73.57	51.07

#### Performance in uncontrolled environment

As shown in Table 11, the LipNet-based 2D-CNN model achieves the highest performance, with an accuracy of 87.15% on Urdu digits and 84.64% on Urdu alphabets. The Hybrid 2D–3D CNN model follows with 82.86 and 78.93%, respectively, while the 3D-CNN model exhibits the lowest performance, achieving 75.72% on digits and 60.89% on alphabets.

The results indicate that the LipNet-based architecture is more effective in handling variability present in uncontrolled environments, likely due to its ability to model temporal dependencies through sequence learning mechanisms. In contrast, the 3D-CNN model, which primarily focuses on local spatiotemporal feature extraction, struggles to capture long-range temporal relationships, resulting in reduced accuracy, particularly for Urdu alphabets.

#### Performance on unseen data

Table 12 evaluates the generalization capability of the models on unseen samples. The LipNet-based model again demonstrates superior performance, achieving 90.72% accuracy on Urdu digits and 81.97% on Urdu alphabets. The Hybrid model achieves comparable performance on digits (90.00%) but shows a notable decline in alphabet recognition (69.45%). The 3D-CNN model performs the worst, with 73.57% on digits and only 51.07% on alphabets.

These results highlight that while some models generalize well for simpler tasks such as digit recognition, their performance degrades significantly for more complex tasks like alphabet recognition. The substantial drop in alphabet accuracy for the Hybrid and 3D-CNN models suggests limited generalization capability and possible overfitting to training data patterns.

#### Digits vs. alphabets recognition

A consistent observation across both tables is that all models perform better on Urdu digits than on Urdu alphabets. The reason is that Urdu digits constitute a smaller and more distinct set of classes, with relatively clear visual articulation patterns. In contrast, Urdu alphabets involve a larger set of classes with significant viseme overlap, where multiple characters share similar lip movements. This overlap increases inter-class similarity, making discrimination more challenging. Additionally, alphabet recognition involves longer and more complex temporal sequences, which further complicates the learning process.

#### Model architecture analysis

The superior performance of the LipNet-based model can be attributed to its integrated approach of combining convolutional feature extraction with recurrent sequence modeling and Connectionist Temporal Classification (CTC) loss. This enables effective alignment between visual features and output sequences, which is particularly important for handling temporal variations in speech.

The Hybrid 2D–3D CNN model, although designed to capture both spatial and temporal features, does not fully exploit sequential dependencies, leading to suboptimal performance, especially on alphabets. The 3D-CNN model, lacking an explicit sequence modeling mechanism, fails to capture long-term dependencies and exhibits the poorest performance across both tasks.

#### Generalization capability

The evaluation on unseen data reveals that the performance gap between digit and alphabet recognition increases, particularly for the Hybrid and 3D-CNN models. This indicates that these models are less capable of generalizing to new speakers or environmental conditions when dealing with complex linguistic units. In contrast, the LipNet-based model maintains relatively stable performance, demonstrating better robustness and generalization.

#### Conclusion

Overall, the experimental results demonstrate that the LipNet-based 2D-CNN model is the most effective architecture for Urdu lip reading tasks in both uncontrolled and unseen scenarios. The findings also emphasize that Urdu alphabet recognition is inherently more challenging than digit recognition due to higher class complexity, viseme similarity, and temporal variability. These challenges necessitate the use of advanced sequence modeling techniques to achieve reliable performance in real-world applications.

### Letters levels analysis of three models through confusion matrices

**Fig. 11:**
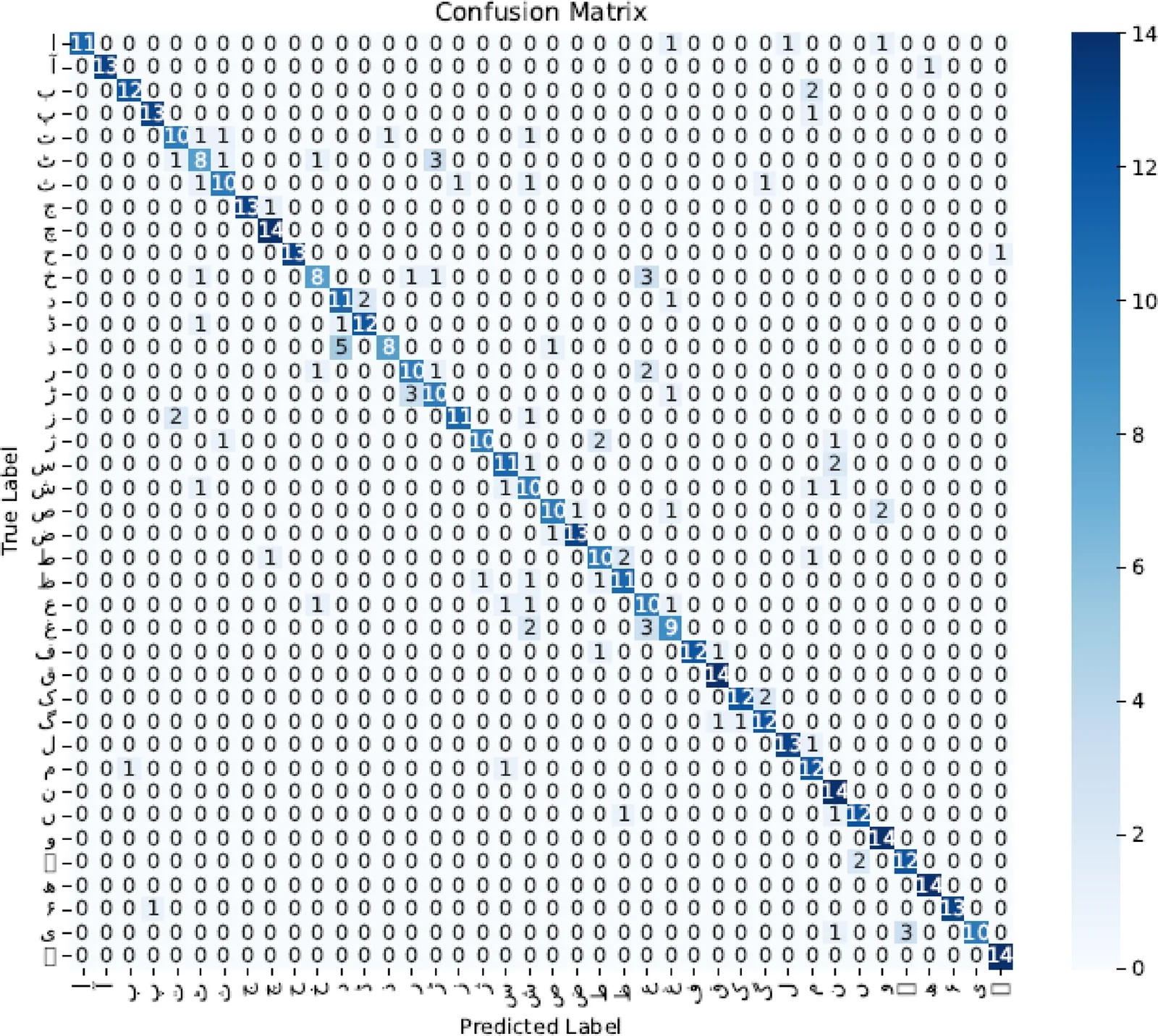
Confusion matrix of 2D-CNN model for letters analysis of urdu alphabet lip reading.

**Fig. 12:**
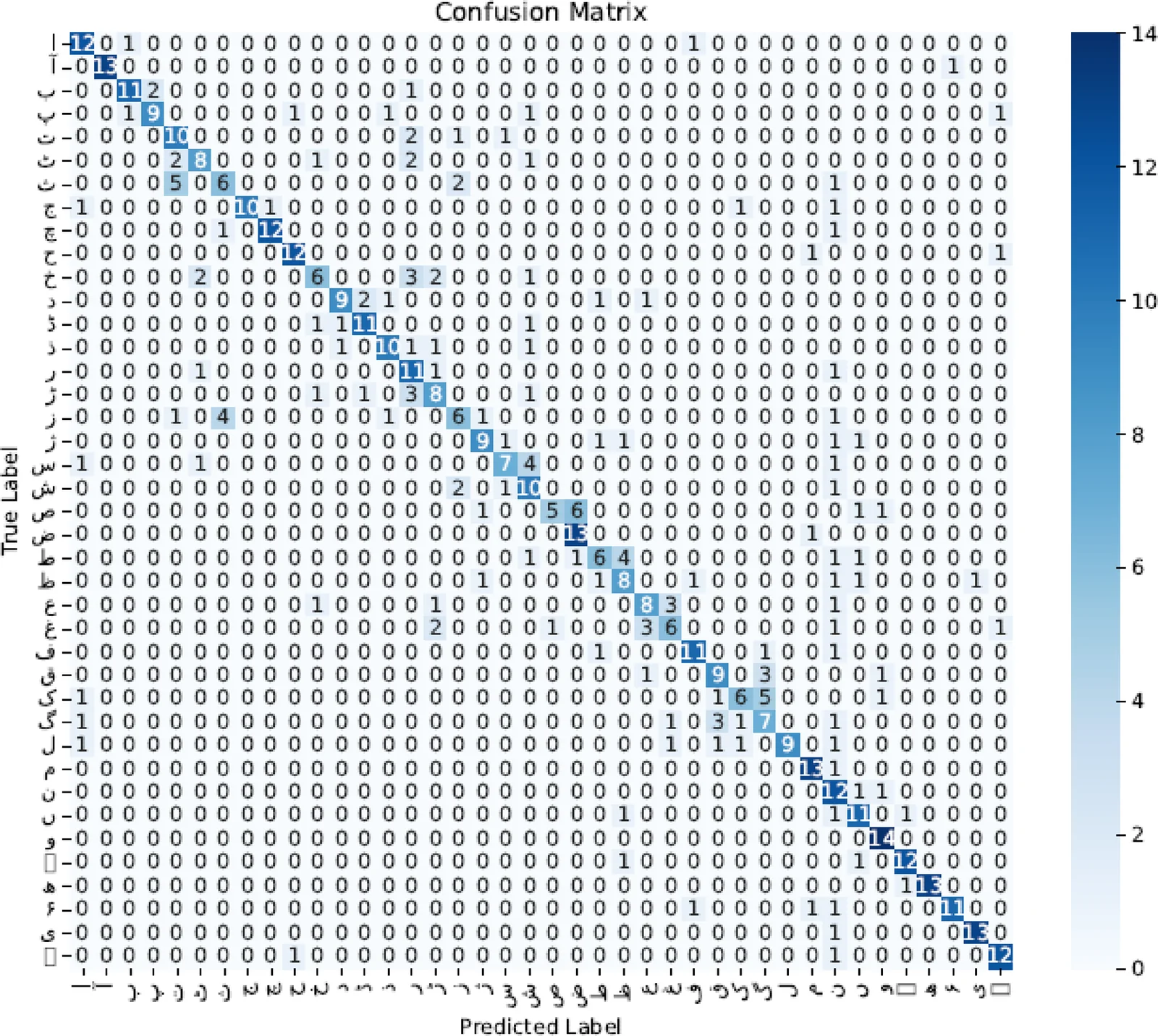
Confusion matrix of hybrid 2D_3D-CNN model for letters analysis of Urdu alphabet lip reading.

**Fig. 13:**
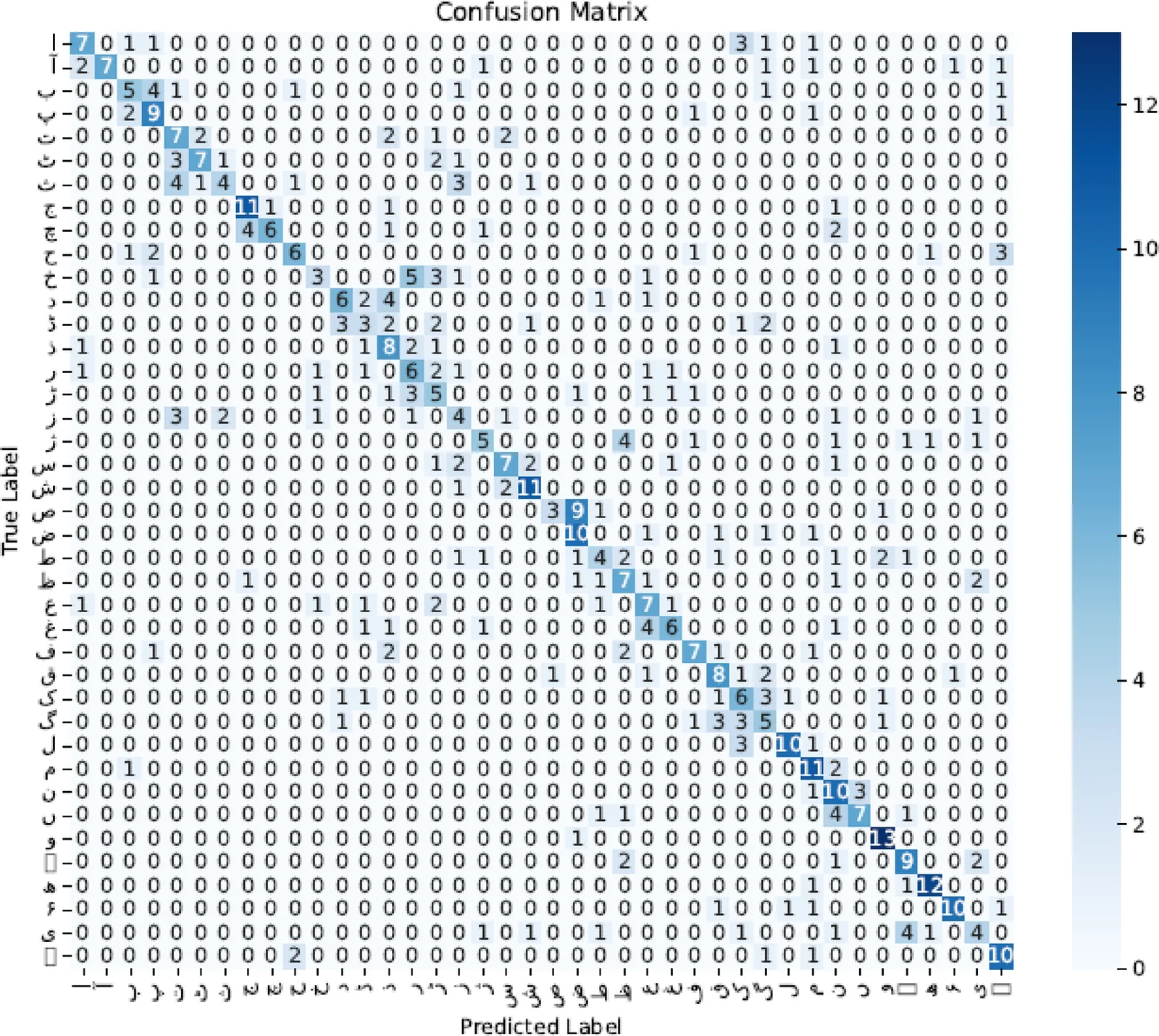
Confusion matrix of 3D-CNN model for letters analysis of Urdu alphabet lip reading.

To analyze all three models at the letter level, confusion matrices are given in Figs. 11, 12 and 13. These matrices indicate the misclassification of true and predicted values. The detailed analysis of each model is as follows.

### Letters level analysis of 2D-CNN Urdu alphabet lip reading model

In Fig. 11, the following are some visually similar pair letters but are misclassified, for example, “خ” and “ع”: 3 instances, it means for 3 different speakers the model predicted the letter ‘’خ as letter ‘ع’, Similarly “د” and “ڈ”: 2 instances, “ت” and “ٹ”: 1 instance, “غ” and “ش”: 2 instances, “ر” and “ع”: 2 instances. Other than these pairs, the detail of misclassified letters is given in the confusion matrix of Fig. 11 and also summarized in Table 13, column 2.

### Letters level analysis of Hybrid 2D_3D-CNN Urdu alphabet lip reading model

In Fig. 12, the following are some visually similar pair letters but are misclassified, for example, “ث” **and** “ت”: 5 instances, it means for 5 different speakers the hybrid model predicted the letter ‘’ث as letter ‘ت’. “ک” and “گ”: 5 instances, “ر” and “ڑ” : 3 instances, “س” and “ش”: 4 instances, “ص” and “ض”: 6 instances, “ک” and “ق”: 3 instances. Other than these pairs, the detail of misclassified letters is given in the confusion matrix of Fig. 12 and also summarized in Table 13, column 3.

### Letters level analysis of 3D-CNN Urdu alphabet lip reading model

In Fig. 13, the following are some visually similar pair letters that are misclassified, for example, letters like " ٹ " and " ت “, " ز " and " ت “, " گ " and " ق”, " گ " and " ک”, " ل " and " ک”, " ک” and” گ “, " ح " and " ے”, " ا” and " ک” : each of these pair misclassification is 3 instances. " د " and " ذ”, " ث” and " ت”, " غ " and " ع”, " ں” and " ن”, " ب " and " پ “, " ز " and " ط”, " ی” and " ے " : each of these pair misclassification is 4 instances. “خ " and “ر " : misclassification is 5 instances. Moreover, the misclassification of 1 and 2 instances between many pairs is also noted. These results show that the model remains very much confused among the majority of pairs. The detail of misclassification of this model is summarized in Table 13, column 4.

Column 2 of Table 13 contains confusion matrix data for the 2D-CNN model. In this model, 58 pairs of letter classes each had a single misclassified instance. Additionally, there were 10, 5, and 1 sets of pairs with 2, 3, and 5 misclassified instances, respectively.

The hybrid 2D_3D-CNN model’s confusion matrix data is presented in column 3 of Table 13. This model had 100 pairs of letter classes, each containing one misclassified instance. There were also 10, 3, 2, 2, and 1 sets of pairs, with each group showing 2, 3, 4, 5, and 1 misclassified instances, respectively.

Confusion matrix data for the 3D-CNN model appears in column 4 of Table 13. This model showed 129 pairs of letter classes, each with a single misclassified instance. Additionally, there were 28, 12, 7, 1, and 1 sets of pairs, each containing 2, 3, 4, 5, and 9 misclassified instances, respectively.

**Table 13:** Letters base classification comparison of three models.

Speaker’s Instances (1)	2D-CNN Model misclassified sets of pairs (2)	Hybrid2D_3D-CNN Model misclassified sets of pairs (3)	3D-CNN Model misclassified sets of pairs (4)	Comparatively best of the three models with fewer misclassified alphabets (5)
1	**58**	100	129	2D-CNN
2	**10**	10	28	2D-CNN
3	5	**3**	12	2D_3D-CNN
4	-	2	7	2D-CNN
5	**1**	2	1	2D-CNN
6	-	1	-	2D-CNN
9	-	-	1	2D-CNN

Column 5 of Table 13 shows that the 2D-CNN model generally performs better, with fewer letter misclassifications than the other two models, except in one instance where the hybrid 2D_3D-CNN model outperformed it. The 3D-CNN model, however, exhibited the poorest performance with significant letter misclassifications, including one set with nine misclassified instances of the same letter.

### Precision, recall, and F1-score analysis of all three models

The confusion matrices are generated from the test data to evaluate the precision, recall, and F1-Score values of all three models. Figures 11, 12, and 13 show the confusion matrices of the LipNet-Based 2D CNN Model, Hybrid 2D-3D CNN Model, and 3D CNN Model respectively.

Using confusion matrices of all three models, shown in Figs. 11, 12, and 13, the following Eqs. (30)–(32) are applied, and precision, recall, and F1-Score values of individual classes are calculated.

30 $$\:Precision=\:\frac{True\:Positives\:\left(TP\right)}{True\:Positivies\:\left(TP\right)+False\:Positives\:\left(FP\right)}\:$$

Where True Positives (TP): Correctly predicted positive cases, and False Positives (FP): incorrectly predicted positive cases.

31 $$\:Recall=\:\frac{True\:Positives\:\left(TP\right)}{True\:Positivies\:\left(TP\right)+False\:Negatives\:\left(FN\right)}\:$$

Where True Positives (TP) are correctly predicted positive cases, and False Negatives (FN) are actual positive cases incorrectly predicted as negative.

32 $$\:F1-Score=\:2\:*\:\frac{Precision*Recall}{Precision+Recall}\:$$

Where true positives (TP) are correctly predicted positive cases, and false negatives (FN) are actual positive cases incorrectly predicted as negative.

By integrating the individual results of Eqs. 30, 31, and 33 of each class in the following Eqs. (33)–(35), Macro averaging values of precision, recall, and F1-Score for multi-class classification are computed.

33 $$\:Macro\:Precision=\:\frac{\sum\:_{i=1}^{n}{Precision}_{i}}{n}\:$$

34 $$\:Macro\:Recall=\:\frac{\sum\:_{i=1}^{n}{Recall}_{i}}{n}$$

35 $$\:Macro\:F1\_Score=\:\frac{\sum\:_{i=1}^{n}{F1\_Score}_{i}}{n}\:$$

Where n is the number of classes, in our case *n* = 40 classes of Urdu alphabets.

Macro averaging precision, recall, and F1-Score of multi-classes of all three models are tabulated in Table 14.

The precision, recall, and F1-Score values of the LipNet-Based 2D CNN Model are high. Based on these results, the LipNet-Based 2D CNN Model is the best of the three models.

**Table 14:** Macro averaging precision, recall, and F1-score values of three lip reading models on unseen data in control and un-control environment.

Multiple models	Results of three test metrics		
	Precision	Recall	F1-score
LipNet-based 2D CNN model	0.83	0.82	0.82
Hybrid 2D-3D CNN model	0.72	0.69	0.69
3D CNN Model	0.54	0.51	0.51

## Conclusion

From the experimental results of training and validation accuracies and training and validation losses of the three models, it is observed that the overfitting issue of the 2D-CNN model was comparatively less than the other two models. The same performance is observed by studying the testing results of these models, here again 2D-CNN model shows better results than its other two competitors by showing 81.97% overall accuracy in diverse environments. On the other hand, when the individual letter base analysis, obtained from the confusion matrix is observed. This study shows that the misclassified letter between true and predicted values of 3D-CNN models was very high, while the hybrid model was at the second level; however, the 2D-CNN model shows the best results in this study. It is concluded that the 2D-CNN model is the best of the three models and is suitable in all respects when tested on known and unknown data, either in diverse environments. Moreover, Table 14 shows that precision, recall, and F1-Score values of LipNet-Based 2D CNN are 0.83, 0.82, and 0.82 respectively. All three values of this model are higher than the other two models. It is concluded that LipNet-Based 2D CNN is the best of the three models in all respects.

## Data Availability

The Urdu Lip Reading Alphabet dataset is uploaded on GitHub platform and is publically available at URL: https://github.com/umair1977/ULRA/blob/main/dataset%20link.
